# Weak noncovalent interactions in two positional isomers of acrylonitrile derivatives: inputs from PIXEL energy, Hirshfeld surface and QTAIM analyses

**DOI:** 10.3389/fchem.2023.1209428

**Published:** 2023-06-28

**Authors:** M. Judith Percino, Mani Udayakumar, Margarita Cerón, Enrique Pérez-Gutiérrez, Perumal Venkatesan, Subbiah Thamotharan

**Affiliations:** ^1^ Instituto de Ciencias, Unidad de Polímeros y Electrónica Orgánica, Benemérita Universidad Autónoma de Puebla, Val3-Ecocampus Valsequillo, Puebla, CP, Mexico; ^2^ Biomolecular Crystallography Laboratory, Department of Bioinformatics, School of Chemical and Biotechnology, SASTRA Deemed University, Thanjavur, India; ^3^ Department of Chemistry, Srimad Andavan Arts and Science College (Autonomous), Tiruchirappalli, India

**Keywords:** acrylonitrile, positional isomers, QTAIM, weak noncovalent interaction, CLP-PIXEL energy, Hirshfeld surface

## Abstract

A single crystal X-ray diffraction analysis was performed on two positional isomers (*m*-tolyl and *p*-tolyl) of acrylonitrile derivatives, namely, (Z)-3-(4-(pyridin-2-yl) phenyl)-2-(m-tolyl) acrylonitrile (**1**) and (Z)-3-(4-(pyridin-2-yl)phenyl)-2-(p-tolyl) acrylonitrile (**2**). Compound **1** crystallized in the monoclinic *P*2_1_/n space group with two crystallographically independent molecules. Compound **2** also possesses two crystallographically independent molecules and crystallized in the triclinic *P*-1 space group. The Hirshfeld surface analysis revealed that, in both isomers, intermolecular H⋅⋅⋅H/C/N contacts contribute significantly to the crystal packing. More than 40% of the contribution arises from intermolecular C–H⋅⋅⋅C(π) contacts. In both compounds, the relative contribution of these contacts is comparable, indicating that the positional isomeric effects are marginal. The structures in which these isomers are arranged in the solid state are very similar, and the lattice energies are also comparable between the isomers. The Coulomb-London-Pauli-PIXEL (CLP-PIXEL) energy analysis identified the energetically significant dimers. The strength of the intra- and intermolecular interactions was evaluated using the quantum theory of atoms in molecules approach. The UV-Vis absorbance in three different solvents (chloroform, ethanol, and ethyl acetate) for isomers **1** and **2** are very similar. This result is in good agreement with the time-dependent density-functional theory (TD-DFT) calculations.

## 1 Introduction

Modern materials and life sciences have shown a great deal of interest in materials based on π-conjugated small organic molecules. Examples of these applications include light-emitting diodes (Friend et al., 1999), sensors (Wang et al., 2013; Gao et al., 2019), photonics (Yan and Evans, 2014), lasers (Gierschner et al., 2016; Kuehne and Gather, 2016; Jiang et al., 2020), photo switches (Feringa and Browne, 2011), sensitizers (Carella et al., 2018) and catalysts (Marzo et al., 2018) as well as bio probes and markers (Tang and Qin, 2013; Wu and Chiu, 2013). In chemistry, the effect of a substituent on the molecular structure of a compound can be explained by several factors, including electronic, steric, and resonance effects. These effects arise due to the presence of substituents and its interactions with the rest of the molecule. The presence of a substituent can alter the distribution of electrons within a molecule, leading to changes in the electronic properties. It is well known that one is the inductive effect, which is based on the electronegativity of the substituent (Mochizuki and Kusama, 2020). An electron-withdrawing group, such as the-CN, tends to withdraw electron density from the rest of the molecule, specifically creating a partial positive charge on the adjacent carbon atom. Conversely, an electron-donating group (alkyl group) can donate electron density, creating a negative charge on the adjacent carbon atom. Furthermore, positional isomerism has been proposed as a molecular design strategy for explaining the inductive effect of functional groups. Additionally, it has provided insight into the strength and nature of intermolecular forces such as van der Waals interactions as well as electronic interactions that modulate the molecular packing of organic materials.

It has been found that intermolecular interactions determine crystal packing with different molecular shapes (Helmers et al., 2020; Monika et al., 2020; Dey et al., 2021). The physicochemical properties of molecules can be determined by investigating how molecules are ordered in relation to their neighbours and how such arrangements are correlated (Forrest and Thompson, 2007; Gierschner and Park, 2013; Hoche et al., 2019). X-ray analysis of single crystals is a common method of determining the molecular arrangement in solids. Structure analysis reveals a wide variety of molecular arrangements for conjugated materials and polymorphs that appear only under special conditions (Percino et al., 2014). This is primarily due to the electronic nature, size, flexibility of the molecular backbone, and position (multiple) of substituents as well as steric demands (Desiraju et al., 2011; Gavezzotti, 2013; Brandenburg and Grimme, 2014; Tiekink, 2014; Beran, 2016). There are numerous factors that influence solid-state fluorescence, including crystal packing (Wang and Li, 2017; Wu et al., 2017), molecular conformation, and noncovalent interactions (Butler et al., 2017; Udayakumar et al., 2019c, 2019b, 2020; Jana et al., 2020). Significant positional effects may occur due to the stabilization of polar structures. There may be large bathochromic shifts caused by ortho and para substituents compared to their corresponding meta isomers. As a result of an electronic effect, dicyano-distyrylbenzenes with cyano groups at the vinyl unit produce twisted geometries due to positional isomers (Gierschner et al., 2021). Computational calculations can provide insight into the nature and strength of the intermolecular forces, such as van der Walls interactions, and electrostatic interactions, which can influence properties of the materials.

In continuation with ongoing interest in the structural and optical properties of acrylonitrile derivatives, we report herein synthesis, optical properties, and single crystal X-ray analysis of the two positional isomers namely, (Z)-3-(4-(pyridin-2-yl) phenyl)-2-(m-tolyl) acrylonitrile (**1**) and (Z)-3-(4-(pyridin-2-yl) phenyl)-2-(p-tolyl) acrylonitrile (**2**). Effect of positional isomers on the molecular conformation, intermolecular interactions, and crystal packing. Different theoretical approaches such as Hirshfeld surface, 2D-fingerprint plots, noncovalent interaction index (NCI) plot, CLP-PIXEL energy and quantum theory of atoms in molecules (QTAIM) and TD-DFT calculations were used to characterize these isomeric compounds.

## 2 Materials and methods

### 2.1 Synthesis

**SCHEME 1:**
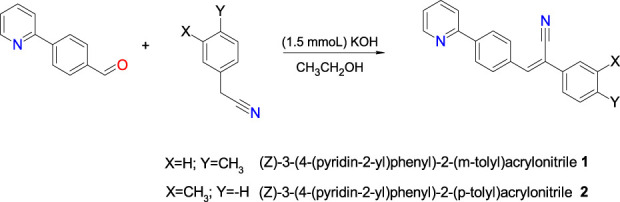
Chemical synthesis of compounds **1** and **2**.

1.5 mmol (0.2715 g) of 4-(2-pyridyl)benzaldehyde was dissolved in 15.0 mL of ethanol and were reacted with 1.5 mmol (0.2 mL) of 3- or 4-methylbenzylcyanide and 1.5 mmol (0.0891 g) of KOH as catalyst (Scheme 1). The reaction was carried out at room temperature for 7 h, until a precipitate was formed, which was filtered and washed with ethanol. Product 1 was purified by recrystallization with ethyl acetate whereas the product 2 was purified with MeOH. The yield 1 was of 53 and 2 of 70% with a melting point of 110°C–112°C, and 160°C–165°C respectively.

### 2.2 Instrumentation

IR spectra of the compounds were recorded on a Vertex 70 FT-IR spectrophotometer (Bruker Optics, Germany) by the diffuse reflectance method. ^1^H and ^13^C NMR spectra were obtained in CDCl_3_ on a Bruker 500 MHz NMR spectrometer. Electron ionization (EI-MS) spectra were acquired on a Joel MStation 700-D mass spectrometer (Joel United States, Peabody, MA). The absorbance spectra (UV-Vis) were acquired with a spectrometer Cary 300 (Agilent Technologies Inc.). FT-IR, ^1^H NMR, ^13^C NMR and mass spectrometry spectra for isomers 1 and 2 in the (Supplementary Figures S1–S6).

#### 2.2.1 (Z)-3-(4-(pyridin-2-yl)phenyl)-2-(m-tolyl)acrylonitrile 1

Colorless block crystals. Yield 53%; mp: 110°C–112°C. ^1^H NMR (CDCl_3_, 500 MHz): δ 8.76–8.75 (d, J = 5 Hz, 1H), 8.15–8.13 (d, J = 10, 5 Hz, 2H), 8.05–8.03 (d, J = 10, 5 Hz, 2H), 7.85–7.82 (d, J = 10, 5 Hz, 2H), 7.61 (s, 1H), 7.54 (s, 1H), 7–54-7–52 (s, J = 10, 5 Hz, 2H), 7.40–7.36 (t, J = 10 Hz, 1H), 7.33–7.30 (q, J = 10, 5 Hz, 1H), 7.26–7.24 (d, J = 10 Hz, 1H), 2.46 (s, 3H). ^13^C NMR (CDCl_3_, 500 MHz): δ 156.22, 149,91, 141.46, 141.10, 138.95, 136.99, 134.39, 134.25, 130.14, 129.79, 129.01, 127.35, 126.76, 123.14, 122.75, 120.83, 118.21, 111.96, 21.54. MS: m/z = 296 [M+] [calcd. for C_21_H_16_N_2_, 296]. FT-IR (KBr) cm^-1^: 3035 (m) (νC-H, A_r_.), 2922, 2863 (w) (ν_s_ C-H, CH_3_), 2214 (ν-C≡N), 1686 (w) (ν-C=C, -CCN = CH-), 1583(s) (ν C=C Ar.). 1464, 1433(m) (δ_as_ -CH_3_), 847 (w) (δ-CH, -CCN = CH-), 752.28, 7884.90 (m) (δ C-H, Ar).

#### 2.2.2 (Z)-3-(4-(pyridin-2-yl)phenyl)-2-(p-tolyl)acrylonitrile 2

Colorless needle crystals. Yield 70%; mp: 160°C–165°C. ^1^H NMR (CDCl_3_, 500 MHz): δ 8.76–8.75 (d, J = 10, 5 Hz, 1H), 8.15–8.12 (d, J = 10, 5 Hz, 2H), 8.05–8.02 (d, J = 10, 5 Hz, 2H), 7.83–7.81 (dd, J = 10, 5 Hz, 2H), 7.64–7.62 (d, J = 10, 5 Hz, 2H), 7.58 (s, 1H), 7.33–7.30 (m, J = 10, 5 Hz, 3H) 2.40 (s, 3H). ^13^C NMR (CDCl_3_, 500 MHz): δ 156.26, 149.90, 140.98, 140.60, 139.57, 136.67, 134.34, 131.64, 129.82, 129.72, 127.34, 125.92, 122.72, 120.81, 118.20, 111.85, 21.31. MS: m/z = 296 [M+] [calcd. for C_21_H_16_N_2,_ 296] FT-IR (KBr) cm^-1^: 3033(m) (νC-H, Ar), 2920, 2864 (ν_s_ C-H, CH_3_), 2216 (ν-C≡N), 1607 (w) (ν-C=C, -CCN = CH-), 1581(s) (ν C=C, Ar).1463(m), 15 (δ_as_ -CH_3_), 847, 817 (w) (δ,-CCN = CH-),), 758, 784 (m) (δ C-H, Ar).

### 2.3 Crystallization

Single crystals 1 were obtained by slow evaporation from a solution of 20 mg dissolved in 1.1 mL of ethyl acetate, which was kept at 4°C for 5 days. Single crystals of 2 were obtained from two different solvents. 10 mg of 2 was dissolved in 2.3 mL of ethyl acetate at 4°C and allowed for slow evaporation. After 9 days, single crystals were harvested for X-ray diffraction analysis. 1.5 mg of 2 was also dissolved in 5 mL of ethanol heated to boiling temperature and after 12–24 h single crystals were appeared suitable for X-ray analysis.

### 2.4 Single crystal X-ray diffraction (SCXRD)

All X-ray intensity measurements were conducted at 110 (2) K. For isomer 1, the X-ray intensities were collected on a SuperNova diffractometer (equipped with Atlas detector) with Cu *K*α (λ = 1.54178 Å) radiation. For isomer 2, two different data sets were collected on an Xcalibur diffractometer (equipped with Sapphire-3 CCD detector) with Mo *K*α (λ = 0.71073 Å) radiation (2i was obtained from ethyl acetate) and on a SuperNova diffractometer (equipped with Atlas detector) with Cu *K*α (λ = 1.54178 Å) radiation (2j was obtained from ethanol). The pre-experiment, data collection, data reduction, and analytical numeric absorption correction (Clark and Reid, 1995) were carried out using the *CrysAlisPro* program (*CrysAlisPro*, version 1.171.36.24, Agilent Technologies). The program *CrysAlisPro* was also used for data reduction and to refine the cell dimensions. The structures were solved by the direct methods with the program *Olex2* (Dolomanov et al., 2009) using SHELXT (Sheldrick, 2015b). The structural refinement was carried out with SHELXL-2018/3 program (Sheldrick, 2015a) by full-matrix least-squares minimization on F^2^. In 1, the pyridyl ring was disordered with two orientations rotated by 180° from one another in both the molecules (A and B). The major disordered components were refined to 0.714 (10) (molecule A) and 0.936 (10) (molecule B). In 2i, H atoms of the methyl group in molecule B were disordered with two sites rotated by 60° from one another. The HFIX 123 option was used to position hydrogen atoms. The occupancy for these hydrogen atoms was fixed at 0.5 with *U*_iso_(H) = 1.5*U*_eq_(C). No disorder was evident in the structure of 2j. The methyl H atoms were constrained to an ideal geometry (C–H = 0.98 Å), with *U*_iso_(H) = 1.5*U*_eq_(C), but they were allowed to rotate freely about the C–C bond. All remaining H atoms were placed in geometrically idealized positions and were constrained to ride on their parent atoms with *U*_iso_(H) = 1.2*U*_eq_(C). The PLATON program (Spek, 2009) was used to check the results of the X-ray analysis, and the MERCURY program (Macrae et al., 2020) was used to render crystal packing and molecular dimers. Due to the disorder of 2i, the structure of 2j was used for all analyses.

### 2.5 DFT calculations

All the DFT calculations were carried out using the program Gaussian-09 program (Frisch et al., 2013) with the M06-2X/cc-pVTZ level of theory (Zhao and Truhlar, 2008) incorporating Grimme’s dispersion correction (D3) (Grimme et al., 2010, 2011). Structural optimization of both monomers of isomers 1 and 2j was performed individually in the gas phase and the major disordered component was used for this calculation. The vibrational frequency calculation using the optimized structures yielded no imaginary frequency indicating they were in minima on their potential energy surface. TD-DFT calculations for both isomers were performed in chloroform solvent using the conductor-like polarizable continuum model (C-PCM) (Cossi et al., 2003). The dimerization energies (Δ*E*_cp_) were calculated using the X-ray geometries with normalized H positions (C–H = 1.083 Å). The values of Δ*E*_cp_ were corrected for basis set superposition error (BSSE) by the counterpoise method (Boys and Bernardi, 1970).

### 2.6 Hirshfeld surface and 2D-fingerprint plots

Hirshfeld surface (HS) analysis was performed to demonstrate the contribution of the various intermolecular interactions formed in the crystal structures. From the Hirshfeld surface, the 2D-fingerprint plots (2D-FP) which correspond to a unique (*d*_e_, *d*_i_) pair. Both HS and 2D-FP were generated using the program CrystalExplorer-17.5 (Spackman et al., 2021).

### 2.7 CLP-PIXEL energy analysis

The intermolecular interaction energies for the molecular dimers and the lattice energies for the crystal structures were calculated using the CLP-PIXEL program (Gavezzotti, 2002, 2003, 2005, 2011). The total energies (intermolecular as well as lattice) were summed by energies of Coulombic (*E*_Coul_), polarization (*E*_pol_), dispersion (*E*_disp_) and repulsion (*E*_rep_) terms. For this calculation, the electron densities for molecules 1 and 2j were calculated at the MP2/6–31G** level of theory (Frisch et al., 1990) using the Gaussian-09 program (Frisch et al., 2013).

### 2.8 QTAIM analysis

The topological properties of the intermolecular interactions observed in the molecular dimers of **1** and **2j** were calculated using the AIMALL package (Keith, 2019). For this calculation, the wavefunctions for the molecular dimers were calculated at their crystal structure geometry with the normalized H positions at the M06-2X-D3/cc-pVTZ level of theory (Zhao and Truhlar, 2008; Grimme et al., 2010, 2011). The dissociation energy (*D*_e_) for the noncovalent interactions was estimated using the EML empirical scheme (Espinosa et al., 1998). The Noncovalent Interaction (NCI) index analysis (Contreras-García et al., 2011) was performed for some of the dimers using the Multiwfn (Lu and Chen, 2012).

## 3 Results and discussion

By a condensation reaction of 4-(2-pyridyl)benzaldehyde with either 3- or 4-methylbenzylcyanide, two tolyl isomers were synthesized and crystal structures have been examined in detail in this work. The molecules have three aromatic rings: pyridyl (ring A), central phenyl (ring B), and methylphenyl (ring C). A methyl group is positioned differently on ring C in compounds **1** (*m*-tolyl) and **2** (*p*-tolyl). These isomers are examined in detail with respect to their molecular conformation, crystal packing, intermolecular interactions and their energetics.

The reaction for the synthesis of **1** and **2** was carried out by Knoevenagel condensation, which is a facile and versatile method for the formation of carbon–carbon bonds. Homogeneous Knoevenagel reactions are normally carried out in the presence of weak bases such as ethylenediamine, piperidine, potassium fluoride, and amino acids (Ryabukhin et al., 2007). Knoevenagel condensations between aldehydes and substrates containing active methylene groups has been carried out in ethanol at room temperature, in the presence of potassium phosphate, to afford unsymmetrical olefins (Zhou et al., 2009; Zhan et al., 2017). The study of compounds **1** and **2**, is important because the condensation reaction has been shown to afford only E-isomers with yields greater than 80% (Al-Shihry, 2004). This could be due to steric effects on the double bond. The melting point of **1** is lower than **2**, which is an indication of the effect of the methyl substituent group in the meta and para positions, respectively. The FT-IR showed that the strong bands in the range of 2214–2216 cm^−1^ which can be assigned for the C≡N stretching. We also noted that these values are comparable with those of reported compounds (Al-Shihry, 2004). The observed FT-IR bands at 2922–2864 and 1680–1680 cm^−1^ which are assigned to C–H and C=C groups, respectively. The EI-MS spectra of **1** and **2** (Supplementary Figures S5, S6), gave a molecular ion peak [M]^+^ at *m/z* 286, which is in agreement with the formula C_21_H_16_N_2_ and molecular mass of 296 g/mol. The spectra with the respective chemical shifts for the synthetized compounds **1** and **2** are shown in Supplementary Figures S3, S4. The singlet signal for the proton of the double bond (-CH = CN) was at the 7.52 and 7.58 ppm for **1** and 2, respectively, indicating a slight effect due to the position of CH_3_ group. From ^13^C spectra, the signals corroborated the formation of both compounds.

### 3.1 Crystal and molecular structures

**FIGURE 1:**
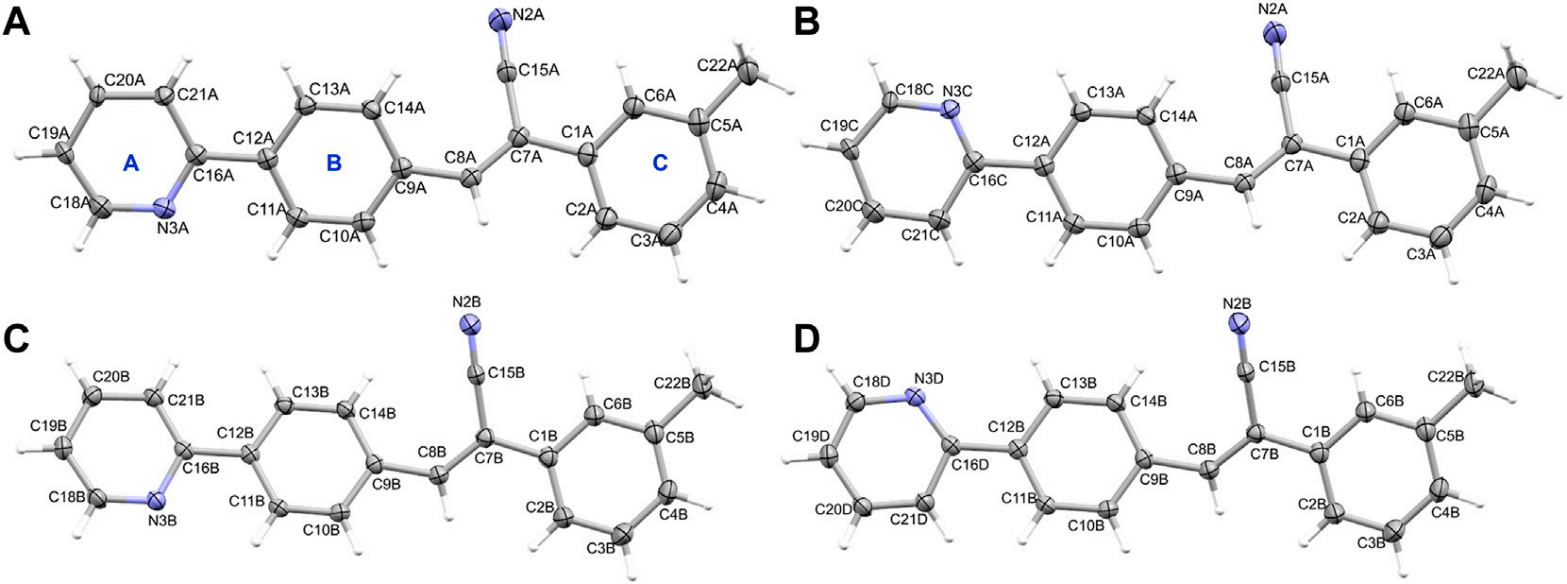
The thermal ellipsoid representation (with 50% probability level) shows the independent view of the asymmetric unit of **1 (A)** molecule A (major disordered component), **(B)** molecule A (minor disordered component), **(C)** molecule B (major disordered component) and **(D)** molecule B (minor disordered component). Rings labels **(A–C)** are indicated for one molecule as a representative.

Crystal data and refinement parameters for compounds **1** and **2** are summarized in Table 1. Compound **1**, namely (Z)-3-(4-(pyridin-2-yl)phenyl)-2-(m-tolyl)acrylonitrile crystallizes in the monoclinic system with the space group P2_1_/n, with two crystallographically independent molecules occupying the asymmetric unit (Z’ = 2). Both molecules (A and B) had disordered pyridyl rings with two different orientations, according to X-ray analysis. In molecule A, the pyridyl ring has a site-occupancy value of 0.714 (10) for the major disordered component and 0.286 (10) for the minor disordered component. The corresponding values for the pyridyl ring in molecule B are 0.936 (10) for the major disordered component and 0.064 (10) for the minor disordered component. In the major disordered components, the pyridyl N atom is positioned relatively in an anti-conformation with respect to the orientation of the cyano (CN) group, while it exhibits a syn conformation in the minor component (Figure 1). Major disordered conformers of molecules A and B are superimposed with the root mean square deviation (RMSD) of 0.21 Å. To further analyze molecules A and B, we considered only their major disordered components. Hereafter, molecules A and B refer to the major disordered components used for analysis.

**TABLE 1:** Crystallographic data and structure refinement parameters of compounds **1** and **2**.

Compound code	1	2i	2j
Empirical formula	C_21_H_16_N_2_	C_21_H_16_N_2_	C_21_H_16_N_2_
Formula weight	296.36	296.36	296.36
Temperature (K)	110 (2)	110 (2)	110 (2)
Crystal system	Monoclinic	Triclinic	Triclinic
Space group	*P*2_1_/n	*P*-1	*P*-1
a (Å)	9.38435 (9)	9.3422 (4)	9.3470 (5)
b (Å)	35.1152 (3)	11.5706 (5)	11.5742 (8)
c (Å)	9.42933 (9)	15.1593 (7)	15.1532 (7)
α (°)	90	90.219 (3)	90.225 (4)
β (°)	99.4298 (9)	93.027 (4)	93.038 (4)
γ (°)	90	111.520 (4)	111.568 (6)
Volume (Å^3^)	3065.29 (5)	1521.81 (12)	1521.92 (16)
Z	8	4	4
ρ_calc_ (g/cm^3^)	1.284	1.293	1.293
μ (mm^−1^)	0.586	0.076	0.590
F (000)	1248	624	624
Crystal size (mm^3^)	0.36 × 0.26 × 0.19	0.53 × 0.11 × 0.09	0.09 × 0.05 × 0.03
Radiation	CuKα (λ = 1.54178)	MoKα (λ = 0.71073)	CuKα (λ = 1.54178)
2Θ range for data collection (°)	9.836 to 143.756	4.58 to 50	5.842 to 143.788
Index ranges	−11 ≤ *h* ≤ 11, −42 ≤ *k* ≤ 43, −11 ≤ *l* ≤ 10	−11 ≤ *h* ≤ 11, −13 ≤ *k* ≤ 13, −18 ≤ *l* ≤ 17	−11 ≤ *h* ≤ 9, −14 ≤ *k* ≤ 14, −18 ≤ *l* ≤ 18
Reflections collected	20028	16485	17887
Independent reflections	6004 [R_int_ = 0.0199, R_sigma_ = 0.0167]	5350 [R_int_ = 0.0466, R_sigma_ = 0.0545]	5973 [R_int_ = 0.0497, R_sigma_ = 0.0547]
Data/restraints/parameters	6004/42/449	5350/0/416	5973/0/417
Goodness-of-fit on F^2^	1.047	1.025	0.982
Final R indexes (I> = 2σ (I))	R_1_ = 0.0354, wR_2_ = 0.0969	R_1_ = 0.0459, wR_2_ = 0.1049	R_1_ = 0.0449, wR_2_ = 0.1021
Final R indexes [all data]	R_1_ = 0.0390, wR_2_ = 0.0999	R_1_ = 0.0821, wR_2_ = 0.1195	R_1_ = 0.0865, wR_2_ = 0.1209
Largest diff. peak/hole (e Å^−3^)	0.25/−0.19	0.26/-0.21	0.20/−0.20
CCDC No.	2256656	2256657	2256658

Molecules A and B of **1** do not exhibit fully planar conformation and there is a slight twist around the acrylonitrile group and ring C with respect to the mean planes of coplanar rings A and B. The dihedral angle is formed between the mean planes of different groups are given in Supplementary Table S1.

**FIGURE 2:**
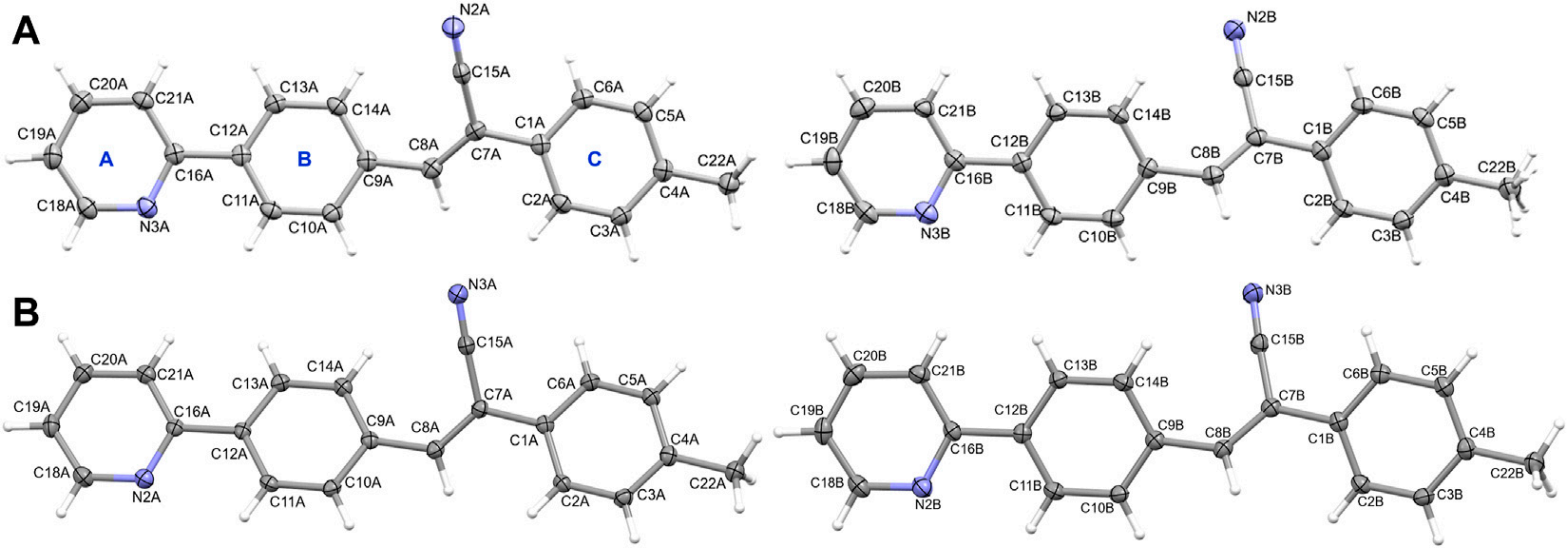
The thermal ellipsoid representation (with 50% probability level) showing the independent view of the asymmetric unit of **(A)** molecules A and B (with disordered methyl H atoms) of **2i** and **(B)** molecules A and B of **2j**. The rings are labelled as **(A–C)**.

Compound **2**, namely (Z)-3-(4-(pyridin-2-yl) phenyl)-2-(p-tolyl) acrylonitrile crystallizes in the triclinic system with the space group P-1. The asymmetric unit contains two crystallographically independent molecules (Z’ = 2), as observed in **1**. The hydrogen atoms of the methyl group of molecule B were disordered in compound 2 (**2i**). The pyridyl N atom also exhibits an anti conformation with respect to the cyano group orientation in this structure. As previously reported acrylonitrile derivatives (Percino et al., 2014; Wang et al., 2022), the pyridyl ring exhibits the same anti-conformation. Figure 2A shows the thermal ellipsoid representation of **2i**. The two independent molecules are superimposed very well with an RMSD of only 0.09 Å for non-hydrogen atoms. During the preparation of this manuscript, we performed another X-ray measurement (CuKα: **2j**) for crystal **2** that produced an ordered structure. Both **2i** and **2j** showed similar cell parameters and similar R-factors. For all analysis used in this work, structure **2j** was used (Figure 2B). It is also noted that the two independent molecules of **2j** are superimposed very well with an RMSD value of 0.09 Å. As observed in **1**, the molecules of A and B of **2j** also do not possess fully planar and there is a slight twist around acrylonitrile and ring C with respect to the mean planes of the coplanar rings A and B (Supplementary Table S2). A comparison of geometrical parameters such as bond lengths and angles between X-ray and optimized structures showed that both are in good agreement (Supplementary Tables S3–S6). The structural overlay diagrams show that they are superimposed very well with the RMSD values of 0.5–0.6 Å and with slight twist on the aromatics rings, suggesting the crystal packing effect (Supplementary Figures S7–S10).

### 3.2 Intramolecular interactions

**FIGURE 3:**
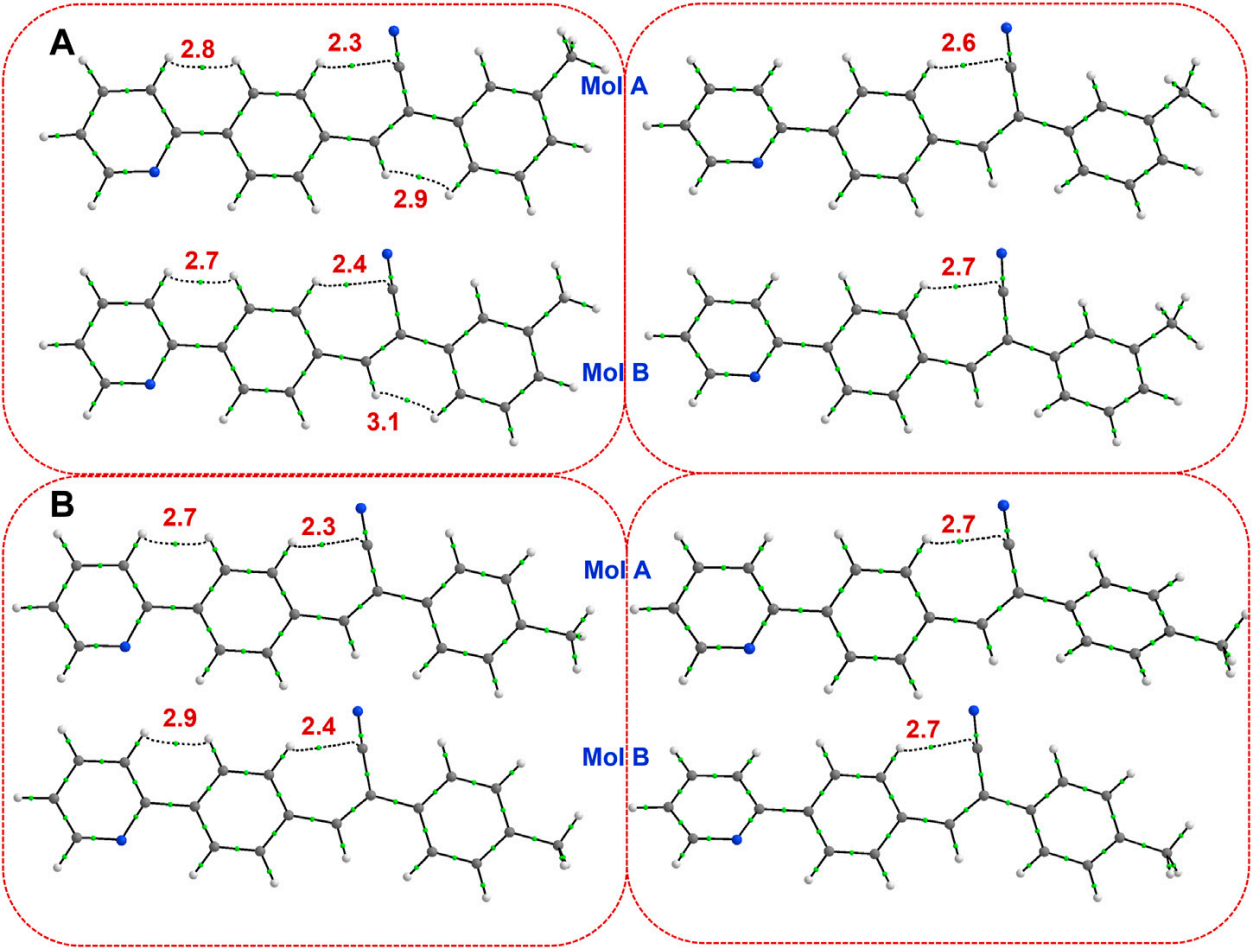
Existence of (3, −1) bond critical points for intramolecular noncovalent interactions in **(A)** isomer **1** (left panel: X-ray and right panel: optimized), and **(B)** isomer **2j** (left panel: X-ray and right panel: optimized). The values correspond to dissociation energy (in kcal mol^-1^).

The QTAIM analysis was performed for both X-ray and optimized structures to study the intramolecular interactions. In **1**, the X-ray conformation (both molecules A and B) shows three intramolecular contacts, of which one of them is the characteristic C–H⋅⋅⋅C contact formed between H atom of ring C and the cyano C15 atom observed in this class of compounds. The remaining two of them are H⋅⋅⋅H contacts (H-H bonding) formed between H atoms of rings A and B and between vinylic CH and H atom of the ring C (Figure 3A). The former H-H bonding and C–H⋅⋅⋅C contacts were observed in closely related structures repored earlier (Venkatesan et al., 2018; Udayakumar et al., 2019c, 2019b, 2020). The importance of the non-electrostatic origin of the H-H bonding has been discussed elsewhere (Matta et al., 2003; Al-Ghulikah et al., 2020; El-Emam et al., 2020). It is also noted that the concept of H-H bonding has also been debated in the literature (Poater et al., 2006). To verify the stability of these intramolecular contacts, we performed structural optimization for both molecules individually. In the optimized structures, both H-H bondings were disappeared and only the characteristic C–H⋅⋅⋅C interactions retained suggesting that H-H bonding help maintaining the planarity of the molecular conformation in the solid state. In the optimized structures, the planarity is slightly twisted and hence H-H bondings are absent. In **2j**, the H-H bonding between vinylic CH and H atom of the ring C disappears due to slight twist of ring C in the X-ray conformation. However, H-H bonding between rings A and B and between H atom of the ring C and the cyano C15 atom are retained in order to maintain the planarity. In the optimized structures of **2j**, only the characteristic C–H⋅⋅⋅C contact was observed. The topological parameters for these intramolecular interactions in X-ray and optimized molecules of **1** and **2j** are summarized in Supplementary Table S7. For the X-ray conformers, the dissociation energy for the H-H bonding between rings A and B is in the range of 2.7–2.9 kcal mol^-1^ and slightly higher (2.9–3.1 kcal mol^-1^) for the H-H bonding between vinylic CH and H atom of ring C. The dissociation energy (2.3–2.4 for X-ray and 2.6–2.7 for optimized structures) for the characteristic C–H⋅⋅⋅C contacts is comparable between X-ray and optimized structures.

### 3.3 Hirshfeld surface (HS) and 2D-Fingerprint plots (2D-FP)

**FIGURE 4:**
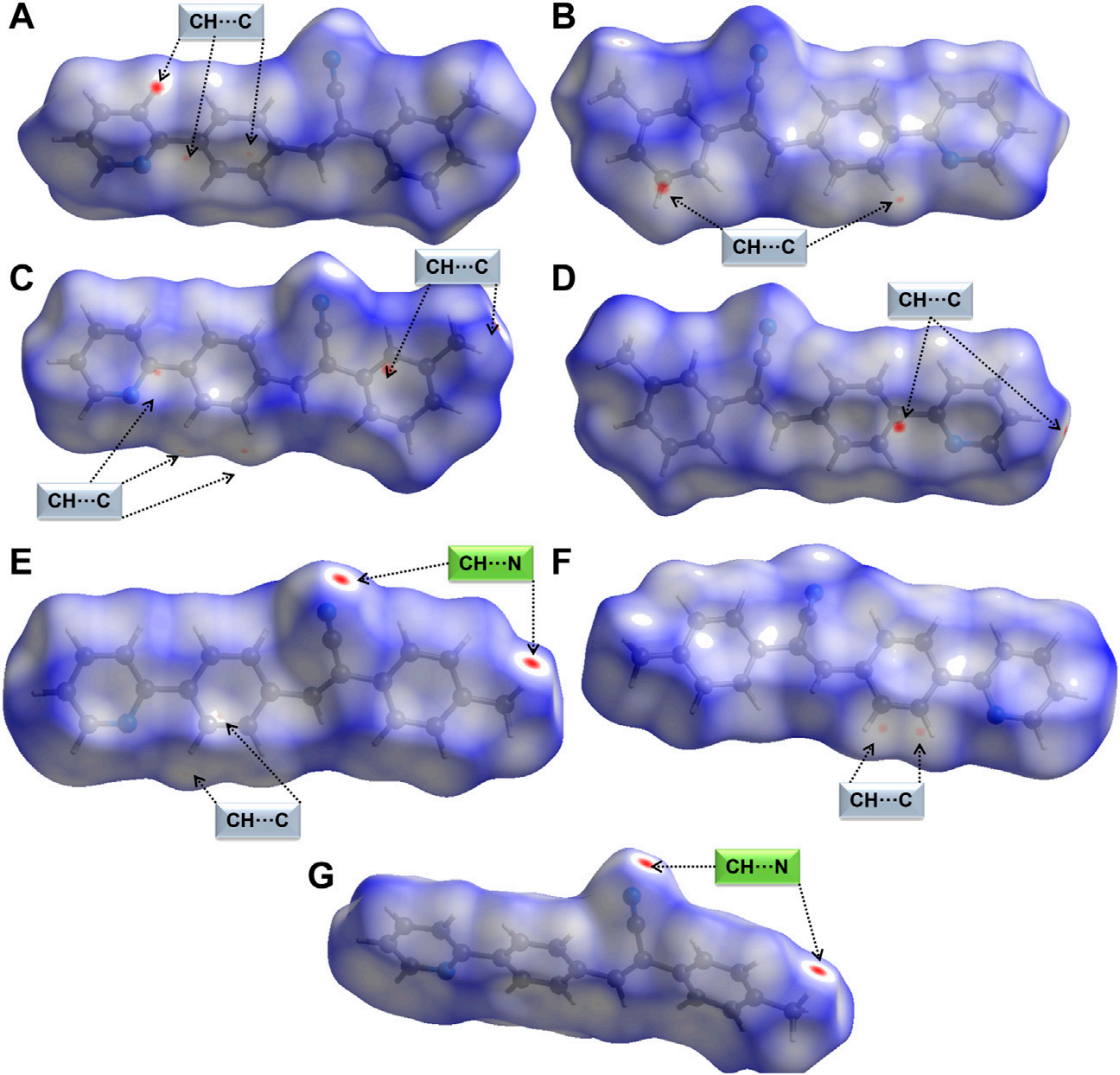
Hirshfeld surfaces are mapped over normalized distances (*d*_norm_) **(A,B)** two different orientations of molecule A of **1**, **(C,D)** two different orientations of molecule B of **1** showing short C–H⋅⋅⋅C(π) interactions, **(E,F)** two different orientations of molecule A of **2j** showing short C–H⋅⋅⋅N and C–H⋅⋅⋅C(π) interactions, and **(G)** molecule B of **2j** showing a pair of C–H⋅⋅⋅N interactions.

Crystal structures have been characterized using HS and 2D-FP to study intermolecular interactions. We used this tool to study the qualitative effect of methyl isomers on intermolecular interactions. The Hirshfeld surface was generated for molecules A and B of **1** and **2j** individually. Figures 4A–D show HS in two different orientations of molecules A and B of **1**. In molecule A, one of the intermolecular C–H⋅⋅⋅C interactions (H21A⋅⋅⋅C12B) shows intense red spots, and the remaining two C–H⋅⋅⋅C interactions (H10B⋅⋅⋅C12A and H11B⋅⋅⋅C14A) display relatively less intense red spots (Figures 4A, B). Molecule B also exhibits similar features to C–H⋅⋅⋅C interactions. The intense red spot is pointing the C⋅⋅⋅C contact which is actually C–H⋅⋅⋅C interaction as characterized by the QTAIM analysis. A similar feature exists in **2j**. Surprisingly, there were no red regions near the cyano N and pyridyl N atoms. This feature suggests that these atoms have weak accepting tendency to participate in the intermolecular interactions.

Figures 4E, F show two different HS orientations mapped over the *d*_norm_ values for molecule A of **2j**. The intense red regions are associated with a pair of intermolecular C–H⋅⋅⋅N interactions and relatively less intense red spots correspond to intermolecular C–H⋅⋅⋅C(π) observed in molecule A (Figures 4E, F). There is a pair of red spots on the surface of molecule B of **2j** corresponding to C–H⋅⋅⋅N interactions (Figure 4G). We also observed bright red spots in similar acrylonitrile derivatives near the cyano N atom that serves as an acceptor for intermolecular interactions (Venkatesan et al., 2018; Udayakumar et al., 2019a, Udayakumar et al., 2019c, Udayakumar et al., 2019b, Udayakumar et al., 2020; Castillo et al., 2023).

Supplementary Figure S11 shows the full and decomposed 2D-FP plots for three different intermolecular contacts (H⋅⋅⋅H, H⋅⋅⋅C and H⋅⋅⋅N) along with their relative contributions to the crystal packing. In **1**, the relative contributions of the above three contacts are comparable between molecules A and B. However, some differences were noticed in the distribution of the respective contacts in the 2D-FP plots. For example, a single spike with a tip distance (*d*_e_ + *d*_i_) of 2.2 Å is observed for H⋅⋅⋅H contacts in molecule A. Contrasting double blunt tips at 2.2 Å are noted for H⋅⋅⋅H contacts in molecule B. The shortest H⋅⋅⋅C contacts which represent intermolecular C–H⋅⋅⋅C(π) interactions observed above 2.7 Å and appear as a typical wing-like pattern in the 2D-FP plot in both molecules A and B. The intermolecular H⋅⋅⋅N contacts shows distinct feature between molecules A and B. In molecule A, the shortest H⋅⋅⋅N distance appears beyond 2.7 Å suggesting weak nature of this contact. In contrast, molecule B shows the short H⋅⋅⋅N distance is less than 2.7 Å indicating a relatively strong nature. The relative contribution of H⋅⋅⋅H, H⋅⋅⋅C and H⋅⋅⋅N contacts are comparable between crystallographically independent molecules of A and B in **2j** and also comparable with the structure **1**. This analysis suggests that the isomeric effect is very marginal with respect to the relative contribution of intermolecular interactions. The decomposed 2D-FP plot shows that similar short H⋅⋅⋅H and H⋅⋅⋅C contacts. However, the H⋅⋅⋅N contact shows the sharp double spikes apparently distinct from molecule A of **1**. The close H⋅⋅⋅N contact is observed around 2.5 Å in both molecules A and B of **2j** which is much less than the sum of the vdW radii of the H and N atoms, suggesting that this contact plays an important role in stabilization.

### 3.4 Molecular dimers and crystal packing of 1

**FIGURE 5:**
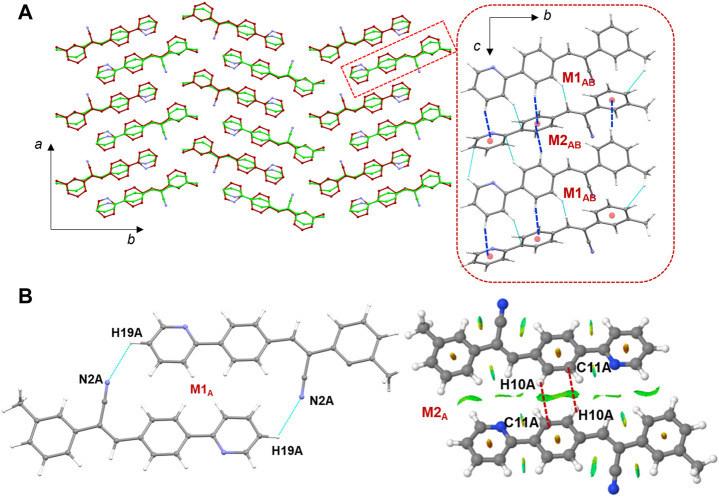
**(A)** Columnar packing of **1** (molecule A: green and molecule B: red) projected onto the crystallographic *ab* plane and a red dashed box indicates the basic structural motif projected onto the *bc* plane (right panel), and **(B)** molecular dimers are formed between molecule A and its symmetry equivalents. The NCI plot analysis shows large patches confirming intermolecular C–H⋅⋅⋅C interactions in dimer M2_A_.

The molecules of **1** are packed in a columnar fashion along the crystallographic *ab* plane. Figure 5A shows the crystal packing of **1** and the basic structural motif (dashed box) formed in this structure. The CLP-PIXEL calculation revealed nine energetically most significant molecular dimers (Table 2). Two, three and four molecular dimers are formed between A (M1_A_ and M2_A_), B (M1_B_-M3_B_) and AB (M1_AB_-M4_AB_) molecules, respectively. The basic structural motif constitutes the alternate motifs of M1_AB_ and M2_AB_ and these motifs are stabilized by intermolecular C–H⋅⋅⋅π interactions (see Figure 5A, dashed box). Regarding the self-association of molecule A and its symmetry equivalent partners, dimer M1_A_ is formed *via* intermolecular C–H⋅⋅⋅N interaction between centrosymmetrically related molecules of A, leading to the generation of a $R_{2}^{2}$ (26) ring motif (Figure 5B). It is noted that this C–H⋅⋅⋅N interaction was established slightly longer than the sum of the vdW radii of the H and N atoms +0.04 Å which is in good agreement with the Hirshfeld surface analysis. The electrostatic and dispersion energies contribute about 44% and 56%, respectively, towards the stabilization of this dimer. On the contrary, the dimer M2_A_ stabilizes with a weak intermolecular C–H⋅⋅⋅C interaction (involving central ring B). Dispersion energy (69%) relatively contributes more to the stabilization of M2_A_. The C–H⋅⋅⋅C angle is below 120°, we further characterized the nature of interaction *via* NCI plot analysis. The appearance of green patches near the interacting regions suggesting weak nature of this interaction (Figure 5B).

**TABLE 2:** Intermolecular interaction energies (in kcal mol^−1^) for different dimers were obtained from the crystal structures of **1** **and 2j using the CLP-PIXEL method. The BSSE-corrected dimerization energies (*ΔE*_cp_) calculated by the DFT method were given for comparison**.

Dimer	CD	Symmetry	Important interactions	Geometry^a^ H···A (Å), ∠D–H···A (°)	PIXEL/MP2/6–31G**					M06-2X-D3/cc-pVTZ
					*E*_Coul_	*E*_pol_	*E*_disp_	*E*_rep_	*E*_tot_	*ΔE*_cp_
**Compound 1**										
**Mol A···Mol A**										
M1_A_	9.365	–x+2, –y+1, –z+2	C19A–H19A···N2A	2.79, 118	−5.6	−2.0	−9.6	6.9	−10.3	−9.8
M2_A_	6.912	–x+1, –y+1, –z+1	C10A–H10A···C11A	2.94, 116	−3.1	−1.1	−9.4	5.3	−8.3	−7.1
**Mol B···Mol B**										
M1_B_	9.427	–x+2, –y+1, –z+1	C19B–H19B···N2B	2.87, 118	−5.2	−1.9	−9.4	6.2	−10.3	−9.5
M2_B_	6.730	–x+1, –y+1, –z	C2B–H2B···C18B	2.90, 122	−3.8	−1.4	−10.5	7.0	−8.8	−7.8
M3_B_	13.796	–x+1, –y+1, –z	C22B–H226···N2B	2.65, 170	−0.9	−0.7	−3.0	2.0	−2.5	−2.3
**Mol A···Mol B/Mol B···Mol A**										
M1_AB_	4.726	x, y, z+1	C13A–H13A···*C*gB	2.77, 130	−3.0	−2.0	−15.5	9.9	−10.6	−11.5
			C14A–H14A···C8B	2.84, 127						
			C20A–H20A···*C*gA	2.75, 128						
			C21A–H21A···C12B	2.72, 133						
			C22A–H222···C4B	2.87, 150						
M2_AB_	4.706	x, y, z	C3A–H3A···*C*gC	2.78, 122	−2.4	−1.9	−15.3	10.4	−9.3	−10.6
			C10A–H10A···*C*gB	2.72, 130						
			C11A–H11A···C16B	2.75, 134						
			C18A–H18A···C19B	2.89, 118						
M3_AB_	8.042	–x+2, –y+1, –z+1	C13B–H13B···C16A	2.80, 123	−3.4	−1.7	−11.7	7.9	−9.0	−9.4
			C14B–H14B···*C*gA	2.91, 119						
			C20B–H20B···C9A	2.86, 118						
			C21B–H21B···*C*gB	2.78, 121						
M4_AB_	6.337	–x+1, –y+1, –z+1	C10B–H10B···C16A	2.78, 134	−2.9	−1.7	−12.9	9.0	−8.5	−9.2
			C11B–H11B···*C*gB	2.73, 121						
**Compound 2j**										
**Mol A···Mol A**										
M1_A_	10.259	–x+1, –y+2, –z+1	C20A–H20A···N3A	2.74, 121	−5.3	−1.9	−8.7	6.1	−9.8	−9.9
M2_A_	6.867	–x, –y+1, –z+1	C10A–H10A···C11A	2.88, 108	−3.4	−1.1	−9.5	5.9	−8.1	−8.0
M3_A_	11.768	–x+1, –y+1, –z	C22A–H22B···N3A	2.52, 161	−4.1	−1.4	−5.2	4.7	−6.1	−6.0
**Mol B···Mol B**										
M1_B_	10.247	–x+2, –y+2, –z+1	C20B–H20B···N3B	2.80, 120	−5.2	−1.9	−8.9	6.0	−10.0	−9.8
M2_B_	6.865	–x+1, –y+1, –z+1	C10B–H10A···C11B	2.94, 107	−3.1	−1.0	−9.5	5.3	−8.4	−7.9
M3_B_	11.774	–x+2, –y+1, –z	C22B–H22E···N3B	2.50, 157	−4.2	−1.6	−5.6	5.3	−6.0	−5.9
** Mol A···Mol B/Mol B···Mol A**										
M1_AB_	4.655	x–1, y, z	C5B–H5B···*C*gC	2.89, 121	−2.6	−1.7	−15.7	9.9	−10.0	−12.0
			C14B–H14B···*C*gB	2.79, 126						
			C21B–H21B···*C*gA	2.87, 136						
M2_AB_	4.692	x, y, z	C2B–H2B···*C*gC	2.88, 122	−1.5	−2.0	−15.8	10.5	−8.7	−10.6
			C10B–H10B···C8A	2.84, 138						
			C11B–H11B···*C*gB	2.72, 128						
			C18B–H18B···*C*gA	2.75, 125						
M3_AB_	5.454	–x+1, –y+1, –z+1	C10A–H10A···*C*gB	2.78, 125	−2.5	−1.6	−13.5	8.9	−8.6	−9.9
			C11A–H11A···C8B	2.76, 133						
			C18A–H18A···*C*gC	2.75, 131						
M4_AB_	10.235	–x+1, –y+1, –z	C22A–H22C···N3B	2.66, 138	−2.9	−1.1	−7.9	5.6	−6.3	−7.5
			C22B–H22F···N3A	2.70, 136						
			C5A–H5A···C7B	2.89, 136						
			C6A–H6A···*C*gC	2,82, 126						
M5_AB_	10.890	–x+1, –y+2, –z+1	C13A–H13A···*C*gA	2.83, 141	−2.3	−0.8	−7.3	4.1	−6.3	−6.9

^a^ Neutron diffraction values are given for all D–H⋅⋅⋅A interactions. *C*gA, *C*gB, and *C*gC are centroids of rings A, B, and C, respectively.

**FIGURE 6:**
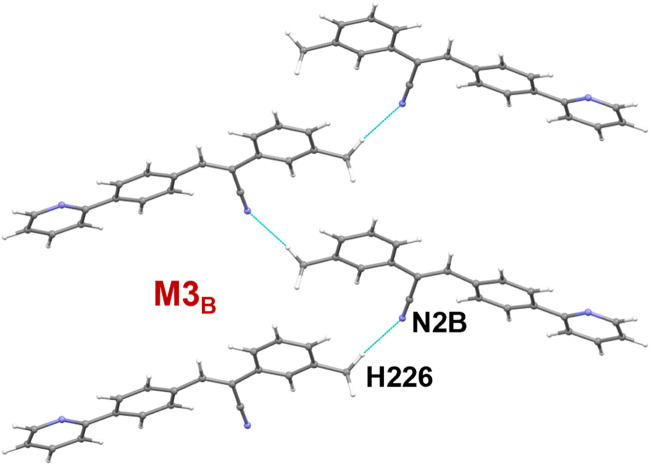
A supramolecular C (8) chain is built using molecule B and its symmetry equivalents using a highly directional intermolecular C–H⋅⋅⋅N interaction in **1**.

It is noted that dimers M1_B_ and M2_B_ are similar to those of M1_A_ and M2_A_ observed in molecule A. The dimeric motifs of M1_B_ and M2_B_ are shown in Supplementary Figure S12. Their intermolecular interaction energies are also comparable. However, the C–H⋅⋅⋅N interaction was established in the M1_B_ dimer is relatively longer than the sum of the vdW radii of the H and N atoms +0.13 Å which is in good agreement with the Hirshfeld surface analysis. The dimer M3_B_ is stabilized by a highly directional intermolecular C–H⋅⋅⋅N interaction (involving methyl group and the cyano N atom) that is specific to molecule B (Figure 6). The dispersion energy contributes approximately 65% to the stabilization of the dimer M3_B_. Furthermore, this interaction links the neighbouring molecules of B into a *C* (8) chain that runs parallel to the crystallographic *a* axis.

**FIGURE 7:**
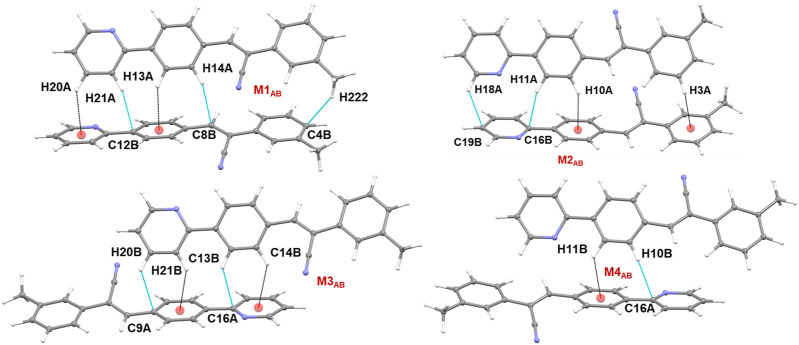
Molecular dimers formed between two crystallographic independent molecules A and B and their symmetry-equivalents in the solid state of **1**.

There are four molecular dimers formed between molecules A and B. These dimers are unusually stabilized by excessive number of C–H⋅⋅⋅π interactions (Figure 7). The intermolecular interaction energies calculated by the PIXEL method range from −10.6 to −9.0 kcal mol^-1^. These energies are comparable to those calculated by the DFT method. The lattice energy calculation indicates that the stabilization of the crystal structure of **1** is primarily driven by the dispersion energy with a contribution of 72% (Supplementary Table S8).

### 3.5 Molecular dimers and crystal packing of 2j

**FIGURE 8:**
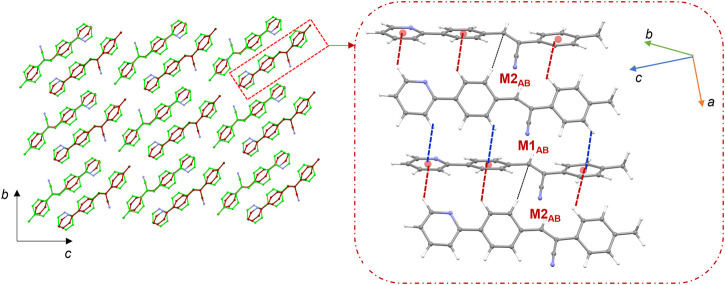
Crystal packing of **2j** projected onto the crystallographic *bc* plane showing the columnar molecular arrangements (molecule A: green and molecule B: brown). The basic structural motif is indicated using dashed boxes.

Figure 8 shows the crystal packing of **2j** and the basic structural motif (dashed box) formed in this structure. The molecules of **2j** are packed in a columnar fashion along the crystallographic *bc* plane. The crystal packing of *m*-tolyl isomer (**1**) is very similar to that of corresponding *p*-tolyl isomer (**2j**). The primary difference between these two crystal packings is the columnar arrangement in different crystallographic planes.

**FIGURE 9:**
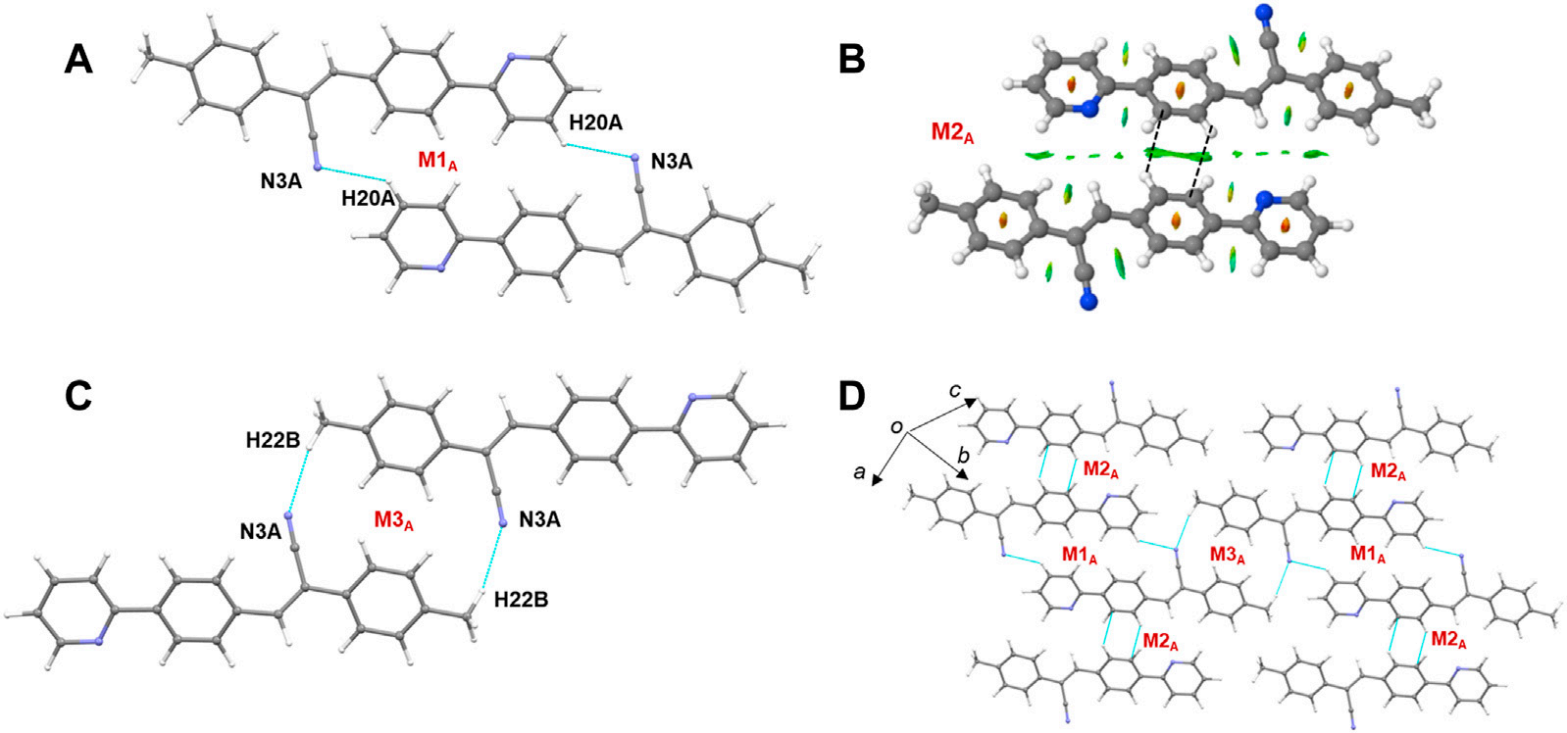
**(A–C)** Molecular dimers are formed between molecule A and its symmetry equivalent partners in **2j** and **(D)** supramolecular sheet built by the motifs M1_A_-M3_A_.

The CLP-PIXEL calculation revealed that at least eleven molecular dimers which are energetically significant formed in the solid state (Table 2). The intermolecular interactions between molecule A and its equivalent molecules of symmetry stabilize three dimers (M1_A_ to M3_A_) *via* C–H⋅⋅⋅N and C–H⋅⋅⋅C(π) interactions (Figures 9A–C). The intermolecular interactions (*E*_tot_) calculated by the CLP-PIXEL method and stabilization energy (Δ*E*_cp_) for molecular dimers calculated by DFT method with M06-2X-D3/cc-pVTZ level of theory are comparable. The centrosymmetrically related molecules of A generate a loop (M1_A_) stabilized by an intermolecular C–H⋅⋅⋅N (involving one of the pyridyl H atom and the cyano N atom) interaction with a graph-set motif of $R_{2}^{2}\left(24\right).$ The electrostatic and dispersion energies contribute 45% and 55%, respectively, to the stabilization of this dimer. The M2_A_ dimer stabilizes with weak intermolecular C–H⋅⋅⋅C(π) contacts as observed in **1**. The NCI plot shows the green patches for the interacting regions suggesting the weak nature (Figure 9B). For the stabilization of this dimer, the dispersion energy contributes about 68%. Furthermore, one of the methyl H atoms acts as a donor for the intermolecular C–H⋅⋅⋅N interaction with the cyano N atom as shown in Figure 9C. As a result of this interaction, two centrosymmetrically related molecules of A form a loop with an $R_{2}^{2}\left(18\right)$ motif (M3_A_). Electrostatic (51%) and dispersion (49%) energies contribute nearly equally to the stabilization of this dimer. As shown in Figure 9D, the alternate motifs of M1_A_ and M3_A_ led to the formation of the molecular ribbon and the adjacent ribbons are interconnected by the M2_A_ motif. Overall, these three dimers cooperatively assembled to form a supramolecular sheet.

Similarly, three dimers (M1_B_ to M3_B_) are formed between molecule B and its symmetry-related partners *via* C–H⋅⋅⋅N and C–H⋅⋅⋅C(π) interactions as observed in molecule A and its symmetry-related equivalents. These three motifs are combined to form a supramolecular sheet which is similar to that of Figure 9D. Furthermore, the intermolecular interaction energies for dimers M1_A_ to M3_A_ are comparable to those of dimers M1_B_ to M3_B_ (Table 2). In addition, the *E*_tot_ and Δ*E*_cp_ values are also comparable.

**FIGURE 10:**
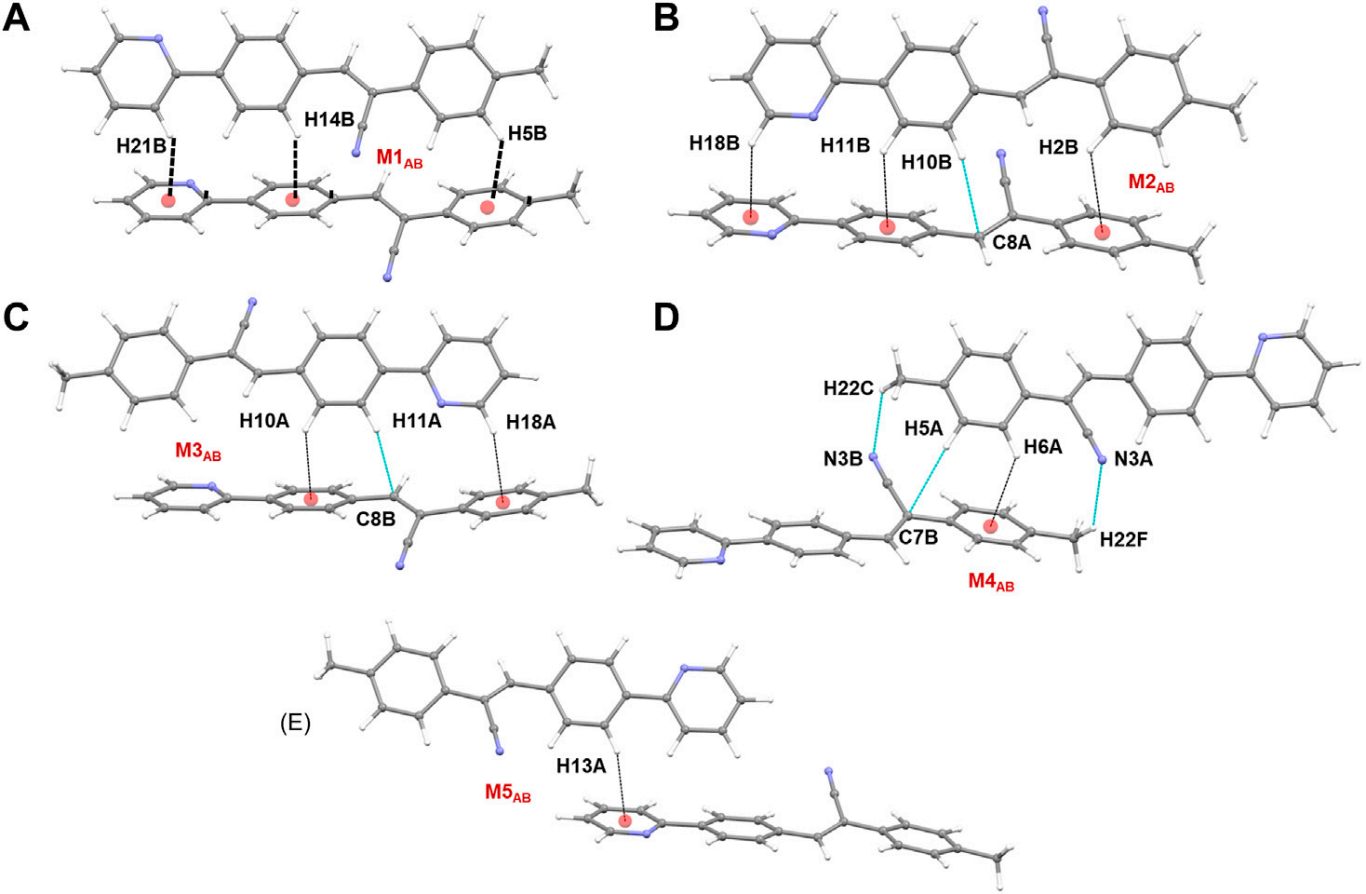
**(A–E)** Molecular dimers are formed between molecules A and B of **2j**. These dimers are stabilized by intermolecular C–H⋅⋅⋅π/N interactions.

Five molecular pairs (M1_AB_ to M5_AB_) are formed between molecules A and B and their symmetry-related counterparts (Figures 10A–E). These five dimers are stabilized by excessive number of C–H⋅⋅⋅π interactions either solely or cooperatively with C–H⋅⋅⋅N interactions. For the stabilization of molecular dimers, the dispersion energies contribute more than the electrostatic energies. It is also noted that the basic structural motif is made up of alternate motifs of M1_AB_ and M2_AB_. Furthermore, we emphasize that only the cyano N acceptor is involved in the interactions and that pyridyl N atom does not participate in the intermolecular interactions could be due to the relatively weak accepting tendency compared to cyano N atom. The crystal lattice energy for **2j** is also comparable to that of **1** suggesting that no effect on the positional isomers (Supplementary Table S8).

### 3.6 Quantitative analysis of intermolecular interactions

The observed interactions in various dimers of isomers **1** and **2j** are further confirmed by the Bader’s atoms in molecules approach (Bader, 1991). The bond critical bonds (BCPs) occur for the mentioned interactions. The molecular graphs for different dimers observed in **1** and **2j** are illustrated in Supplementary Figures S13, S14. The topological parameters for the intermolecular interactions in dimers of **1** and **2j** are summarized in Supplementary Table S9. The result suggests that all observed interactions are closed shell in nature as judged by | $\frac{-V\left(r\right)}{G\left(r\right)}$ | < 1.0 (V(r) is the total potential electronic energy density and G(r) is the total kinetic electronic energy density). In 1, the dissociation energies (*D*_e_) for C–H⋅⋅⋅N interactions are in the range of 0.73–1.00 kcal mol^−1^ and the corresponding energies for C–H⋅⋅⋅π interactions are in the range of 0.71–1.21 kcal mol^−1^. It clearly shows that some of the C–H⋅⋅⋅π interactions are relatively stronger than C–H⋅⋅⋅N interactions. The contrasting feature is noted in **2j** i.e., the strength of the C–H⋅⋅⋅N interactions (1.40–1.47 kcal mol^−1^) is relatively stronger than C–H⋅⋅⋅π interactions (0.68–1.03 kcal mol^−1^).

### 3.7 UV-vis absorption properties

**FIGURE 11:**
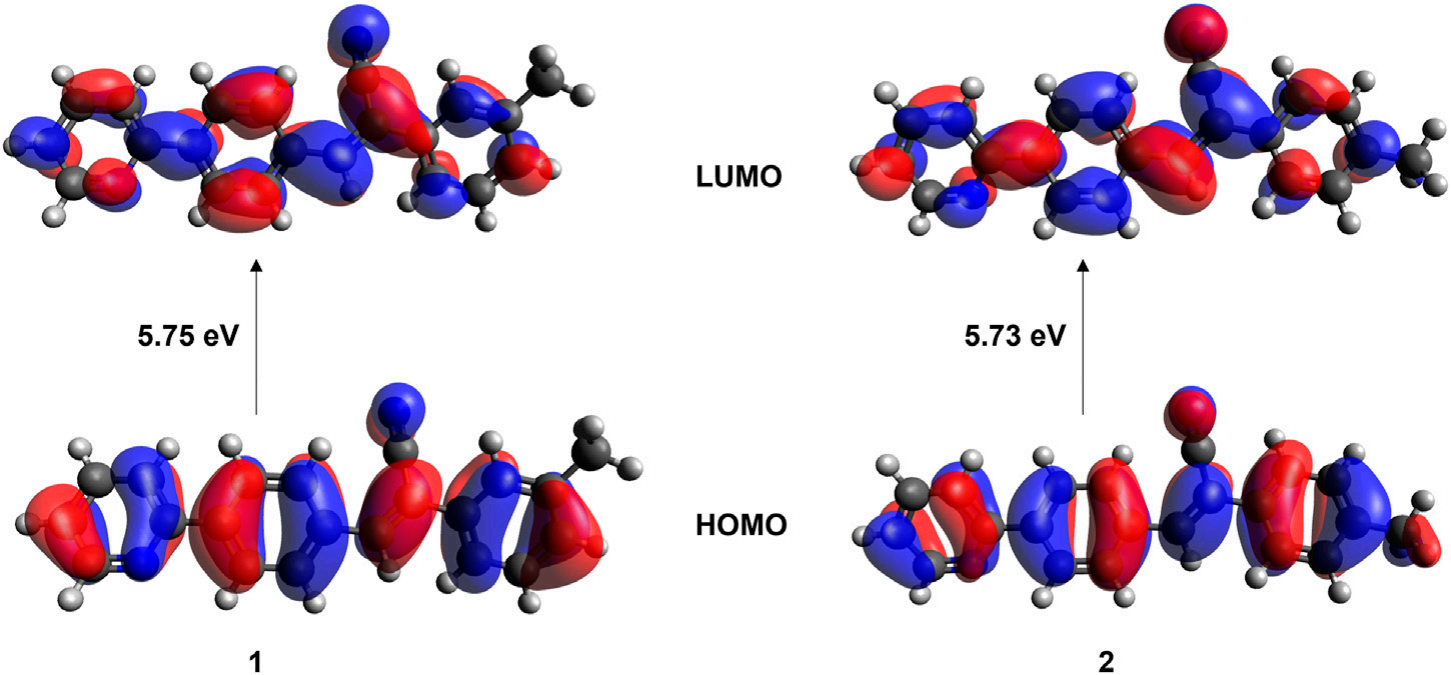
Localization of HOMO and LUMO molecular orbitals in isomers **1** and **2**.

The absorption spectra for **1** and **2** were measured in three different solvents (chloroform, ethanol, and ethyl acetate) and in the solid state to understand the isomeric effect of *m*- and *p*-tolyl groups on optical properties (Supplementary Figures S15–S18). The observed λ_max_ in different solvents are summarized in Table 3. The observed λ_max_ peaks could be assigned to the π → π* transition. The results suggest that solvents do not influence the absorption maxima. This is because *m*- and *p*-tolyl moieties do not alter the absorption behaviours in solution. Further, the optical bandgap energy for **1** and **2** was calculated from the experimental UV absorption spectrum in chloroform solvent via Tauc’s plot (Tauc, 1968). As shown in Supplementary Figure S19, the optical bandgap energy calculated by the Tauc’s plot method showed comparable values for **1** (3.15 eV) and **2** (3.18 eV). In order to corroborate the experimental data by the theoretical calculation using the TD-DFT approach at the M06-2X/cc-pVTZ level of theory, we used chloroform as a representative solvent. The results indicate that the observed and simulated absorption maxima are comparable to the difference of 12–13 nm, which is in the acceptable range. Solid-state UV-vis absorption spectra show that both compounds display the same λ_max_ value that is red-shifted compared to the solution phase. This red shift could be due to the presence of intermolecular interactions. Moreover, structural analysis suggests that both compounds exhibit similar packing and similar types of intermolecular interactions in the solid state, thus one can expect similar optical property. We also investigated the electron localization in the HOMO (highest occupied molecular orbital) and LUMO (least unoccupied molecular orbitals) molecular orbitals. As expected from the crystal structure point of view, the localization of the HOMO and LUMO electron densities showed similar features in both isomers (Figure 11). The bandgap energy between HOMO and LUMO orbitals is also very similar between isomers which is in good agreement with the values calculated by Tauc’s plot. It is also noted that the bandgap energy value is very similar to that of closely related structures reported earlier (Udayakumar et al., 2020).

**TABLE 3:** Excitation wavelengths (in nm), configuration, and oscillator strengths for the isomers **1** and **2**.

Isomer	Solvent	Main Configuration (%)	TD-DFT (in CHCl_3_) at M06-2X/cc-pVTZ					Experimental (λ_max_)	
			λ_max_	*f*	HOMO (eV)	LUMO (eV)	ΔE (eV)	Solution	Solid
**1**	Chloroform	H → L (96)	328	1.394	−7.35	−1.60	5.75	341	394
	Ethanol							336	
	Ethyl acetate							338	
**2**	Chloroform	H → L (96)	331	1.432	−7.30	−1.57	5.73	343	394
	Ethanol							340	
	Ethyl acetate							338	

## 4 Conclusion

Two positional isomers (*m*-tolyl and *p*-tolyl) of the acrylonitrile derivatives were synthesized, and these compounds were characterized using FT-IR, 1H-NMR, EI mass spectrometry, UV-vis absorption, and single crystal X-ray diffraction methods. X-ray analysis revealed that both isomers exhibit very similar molecular arrangement and crystal packing in the solid state, suggesting that isomeric effect is very marginal. Both isomers also showed similar lattice energies calculated by the CLP-PIXEL method. This similarity was also reflected in the solid-state absorbance and had similar λ_max_ values. The UV-vis absorption in three different solvents (chloroform, ethanol and ethyl acetate) was also showed similar λ_max_ values suggesting that the solvents do not influence the optical properties much and the isomeric effect was also very marginal. Hirshfeld surface and 2D fingerprint plots revealed the contribution of important intermolecular interactions help stabilizing the crystal structure and nature of these contacts, respectively. The CLP-PIXEL energy analysis identified the energetically significant molecular dimers observed in these isomers. The structure analysis showed that unusually excessive number of C–H⋅⋅⋅π interactions formed between crystallographically independent molecules in both cases. Finally, we also characterized the strength of intermolecular C–H⋅⋅⋅N/π interactions using the topological parameters. The results showed some difference between isomers.

## Data Availability

The supplementary crystallographic data could be obtained free of charge from Cambridge Crystallographic Data Centre (www.ccdc.cam.ac.uk/data_request/cif) using the accession numbers, CCDC-2256656 (1), CCDC-2256657 (2i), and CCDC-2256658 (2j).

## References

[B1] Al-Ghulikah H. A. Gopalan A. Vahisan L. P. S. Khalaf M. A. Ghabbour H. A. El-Emam A. A. (2020). Insights into the weak Csp3–H···H–Csp3 mediated supramolecular architecture in ethyl 2-(5-bromopentanamido)-4,5,6,7-tetrahydrobenzo[b]thiophene-3-carboxylate, a probable selective COX-2 lead molecule: An integrated crystallographic and theoretical approach. J. Mol. Struct. 1199, 127019. 10.1016/j.molstruc.2019.127019

[B2] Al-Shihry S. S. (2004). Synthesis of substituted stilbenes via the Knoevenagel condensation. Molecules 9, 658–665. 10.3390/90800658 18007467 PMC6147280

[B3] Bader R. F. W. (1991). A quantum theory of molecular structure and its applications. Chem. Rev. 91, 893–928. 10.1021/cr00005a013

[B4] Beran G. J. O. (2016). Modeling polymorphic molecular crystals with electronic structure theory. Chem. Rev. 116, 5567–5613. 10.1021/acs.chemrev.5b00648 27008426

[B5] Boys S. F. Bernardi F. (1970). The calculation of small molecular interactions by the differences of separate total energies. Some procedures with reduced errors. Mol. Phys. 19, 553–566. 10.1080/00268977000101561

[B6] Brandenburg J. G. Grimme S. (2014). Accurate modeling of organic molecular crystals by dispersion-corrected density functional tight binding (DFTB). J. Phys. Chem. Lett. 5, 1785–1789. 10.1021/jz500755u 26273854

[B7] Butler T. Wang F. Sabat M. Fraser C. L. (2017). Controlling solid-state optical properties of stimuli responsive dimethylamino-substituted dibenzoylmethane materials. Mater. Chem. Front. 1, 1804–1817. 10.1039/C7QM00157F

[B8] Carella A. Borbone F. Centore R. (2018). Research progress on photosensitizers for DSSC. Front. Chem. 6, 481. 10.3389/fchem.2018.00481 30364239 PMC6193062

[B9] Castillo A. E. Pérez-Gutiérrez E. Ceballos P. Venkatesan P. Thamotharan S. Siegler M. A. (2023). Non-covalent interactions towards 2-(4-(2,2-dicyanovinyl) benzylidene)malononitrile packing polymorphism due to solvent effect. Experimental and theoretical spectroscopy approach. J. Mol. Struct. 1275, 134674. 10.1016/j.molstruc.2022.134674

[B10] Clark R. C. Reid J. S. (1995). The analytical calculation of absorption in multifaceted crystals. Acta Crystallogr. Sect. A 51, 887–897. 10.1107/S0108767395007367

[B11] Contreras-García J. Johnson E. R. Keinan S. Chaudret R. Piquemal J.-P. Beratan D. N. (2011). Nciplot: A program for plotting non-covalent interaction regions. J. Chem. Theory Comput. 7, 625–632. 10.1021/ct100641a 21516178 PMC3080048

[B12] Cossi M. Rega N. Scalmani G. Barone V. (2003). Energies, structures, and electronic properties of molecules in solution with the C-PCM solvation model. J. Comput. Chem. 24, 669–681. 10.1002/jcc.10189 12666158

[B13] Desiraju G. R. Vittal J. J. Ramanan A. (2011). Crystal engineering. Bangalore, India: Co-Published with Indian Institute of Science IISc. 10.1142/8060 :

[B14] Dey A. Ramlal V. R. Sankar S. S. Kundu S. Mandal A. K. Das A. (2021). Self-assembled cationic organic nanosheets: Role of positional isomers in a guanidinium-core for efficient lithium-ion conduction. Chem. Sci. 12, 13878–13887. 10.1039/D1SC04017K 34760173 PMC8549776

[B15] Dolomanov O. V. Bourhis L. J. Gildea R. J. Howard J. A. K. Puschmann H. (2009). OLEX2: A complete structure solution, refinement and analysis program. J. Appl. Crystallogr. 42, 339–341. 10.1107/S0021889808042726

[B16] El-Emam A. A. Saveeth Kumar E. Janani K. Al-Wahaibi L. H. Blacque O. El-Awady M. I. (2020). Quantitative assessment of the nature of noncovalent interactions in N-substituted-5-(adamantan-1-yl)-1,3,4-thiadiazole-2-amines: Insights from crystallographic and QTAIM analysis. RSC Adv. 10, 9840–9853. 10.1039/D0RA00733A 35498588 PMC9050220

[B17] Espinosa E. Molins E. Lecomte C. (1998). Hydrogen bond strengths revealed by topological analyses of experimentally observed electron densities. Chem. Phys. Lett. 285, 170–173. 10.1016/S0009-2614(98)00036-0

[B18] Feringa B. L. Browne W. R. (2011). Molecular switches. John Wiley & Sons.

[B19] Forrest S. R. Thompson M. E. (2007). Introduction: organic electronics and optoelectronics. Chem. Rev. 107, 923–925. 10.1021/cr0501590

[B20] Friend R. H. Gymer R. W. Holmes A. B. Burroughes J. H. Marks R. N. Taliani C. (1999). Electroluminescence in conjugated polymers. Electroluminescence conjugated Polym. 397, 121–128. 10.1038/16393

[B21] Frisch M. J. Head-Gordon M. Pople J. A. (1990). A direct MP2 gradient method. Chem. Phys. Lett. 166, 275–280. 10.1016/0009-2614(90)80029-D

[B22] Frisch M. J. Trucks G. W. Schlegel H. B. Scuseria G. E. Robb M. A. Cheeseman J. R. (2013). Gaussian 09, revision D.01. Wallingford, CT, USA: Gaussian Inc.

[B23] Gao R. Fang X. Yan D. (2019). Recent developments in stimuli-responsive luminescent films. J. Mater. Chem. C 7, 3399–3412. 10.1039/C9TC00348G

[B24] Gavezzotti A. (2002). Calculation of intermolecular interaction energies by direct numerical integration over electron densities. I. Electrostatic and polarization energies in molecular crystals. J. Phys. Chem. B 106, 4145–4154. 10.1021/jp0144202

[B25] Gavezzotti A. (2003). Calculation of intermolecular interaction energies by direct numerical integration over electron densities. 2. An improved polarization model and the evaluation of dispersion and repulsion energies. J. Phys. Chem. B 107, 2344–2353. 10.1021/jp022288f

[B26] Gavezzotti A. (2005). Calculation of lattice energies of organic crystals: The PIXEL integration method in comparison with more traditional methods. Z. für Krist. - Cryst. Mater. 220, 499–510. 10.1524/zkri.220.5.499.65063:

[B27] Gavezzotti A. (2011). Efficient computer modeling of organic materials. The atom–atom, Coulomb–London–Pauli (AA-CLP) model for intermolecular electrostatic-polarization, dispersion and repulsion energies. New J. Chem. 35, 1360–1368. 10.1039/C0NJ00982B

[B28] Gavezzotti A. (2013). The “sceptical chymist”: Intermolecular doubts and paradoxes. CrystEngComm 15, 4027–4035. 10.1039/C3CE00051F

[B29] Gierschner J. Park S. Y. (2013). Luminescent distyrylbenzenes: Tailoring molecular structure and crystalline morphology. J. Mater. Chem. C 1, 5818–5832. 10.1039/C3TC31062K

[B30] Gierschner J. Varghese S. Park S. Y. (2016). Organic single crystal lasers: A materials view. Adv. Opt. Mater. 4, 348–364. 10.1002/adom.201500531

[B31] Gierschner J. Shi J. Milián-Medina B. Roca-Sanjuán D. Varghese S. Park S. (2021). Luminescence in crystalline organic materials: From molecules to molecular solids. Adv. Opt. Mater. 9, 2002251. 10.1002/adom.202002251

[B32] Grimme S. Antony J. Ehrlich S. Krieg H. (2010). A consistent and accurate *ab initio* parametrization of density functional dispersion correction (DFT-D) for the 94 elements H-Pu. J. Chem. Phys. 132, 154104. 10.1063/1.3382344 20423165

[B33] Grimme S. Ehrlich S. Goerigk L. (2011). Effect of the damping function in dispersion corrected density functional theory. J. Comput. Chem. 32, 1456–1465. 10.1002/jcc.21759 21370243

[B34] Helmers I. Shen B. Kartha K. K. Albuquerque R. Q. Lee M. Fernández G. (2020). Impact of positional isomerism on pathway complexity in aqueous media. Angew. Chem. Int. Ed. 59, 5675–5682. 10.1002/anie.201911531 PMC7154731 31849157

[B35] Hoche J. Schulz A. Dietrich L. M. Humeniuk A. Stolte M. Schmidt D. (2019). The origin of the solvent dependence of fluorescence quantum yields in dipolar merocyanine dyes. Chem. Sci. 10, 11013–11022. 10.1039/C9SC05012D 32206253 PMC7069518

[B36] Jana P. Paramasivam M. Khandelwal S. Dutta A. Kanvah S. (2020). Perturbing the AIEE activity of pyridine functionalized α-cyanostilbenes with donor substitutions: An experimental and DFT study. New J. Chem. 44, 218–230. 10.1039/C9NJ03693H

[B37] Jiang Y. Liu Y.-Y. Liu X. Lin H. Gao K. Lai W.-Y. (2020). Organic solid-state lasers: A materials view and future development. Chem. Soc. Rev. 49, 5885–5944. 10.1039/D0CS00037J 32672260

[B38] Keith T. A. (2019). AIMAll, ver. 19.02.13 Overland Park, KS: TK Gristmill Software.

[B39] Kuehne A. J. C. Gather M. C. (2016). Organic lasers: Recent developments on materials, device geometries, and fabrication techniques. Chem. Rev. 116, 12823–12864. 10.1021/acs.chemrev.6b00172 27501192

[B40] Lu T. Chen F. (2012). Multiwfn: A multifunctional wavefunction analyzer. J. Comput. Chem. 33, 580–592. 10.1002/jcc.22885 22162017

[B41] Macrae C. F. Sovago I. Cottrell S. J. Galek P. T. A. McCabe P. Pidcock E. (2020). Mercury 4.0: From visualization to analysis, design and prediction. J. Appl. Crystallogr. 53, 226–235. 10.1107/S1600576719014092 32047413 PMC6998782

[B42] Marzo L. Pagire S. K. Reiser O. König B. (2018). Visible-light photocatalysis: Does it make a difference in organic synthesis? Angew. Chem. Int. Ed. 57, 10034–10072. 10.1002/anie.201709766 29457971

[B43] Matta C. F. Hernández-Trujillo J. Tang T.-H. Bader R. F. W. (2003). Hydrogen–hydrogen bonding: A stabilizing interaction in molecules and crystals. Chem. – A Eur. J. 9, 1940–1951. 10.1002/chem.200204626 12740840

[B44] Mochizuki H. Kusama H. (2020). A slight bluish-white fluorescence from E,E-2,6-bis(4-cyanostyryl)pyridine pristine crystals. RSC Adv. 10, 2727–2733. 10.1039/C9RA09576D 35496115 PMC9048612

[B45] MonikaVerma A. Tiwari M. K. Show B. Saha S. (2020). Modulation of weak interactions in structural isomers: Positional isomeric effects on crystal packing and physical properties and solid-state thin-film fabrication. ACS Omega 5, 448–459. 10.1021/acsomega.9b02962 31956791 PMC6964309

[B46] Percino M. J. Cerón M. Ceballos P. Soriano-Moro G. Castro M. E. Chapela V. M. (2014). Important role of molecular packing and intermolecular interactions in two polymorphs of (Z)-2-phenyl-3-(4-(pyridin-2-yl)phenyl)acrylonitrile. Preparation, structures, and optical properties. J. Mol. Struct. 1078, 74–82. 10.1016/j.molstruc.2014.04.088

[B47] Poater J. Solà M. Bickelhaupt F. M. (2006). Hydrogen–hydrogen bonding in planar biphenyl, predicted by atoms-in-molecules theory, does not exist. Chem. – A Eur. J. 12, 2889–2895. 10.1002/chem.200500850 16528767

[B48] Ryabukhin S. V. Plaskon A. S. Volochnyuk D. M. Pipko S. E. Shivanyuk A. N. Tolmachev A. A. (2007). Combinatorial Knoevenagel reactions. J. Comb. Chem. 9, 1073–1078. 10.1021/cc070073f 17900167

[B49] Sheldrick G. (2015a). Crystal structure refinement with SHELXL. Acta Crystallogr. Sect. C 71, 3–8. 10.1107/S2053229614024218 PMC4294323 25567568

[B50] Sheldrick G. (2015b). SHELXT - integrated space-group and crystal-structure determination. Acta Crystallogr. Sect. A 71, 3–8. 10.1107/S2053273314026370 PMC4283466 25537383

[B51] Spackman P. R. Turner M. J. McKinnon J. J. Wolff S. K. Grimwood D. J. Jayatilaka D. (2021). CrystalExplorer: A program for Hirshfeld surface analysis, visualization and quantitative analysis of molecular crystals. J. Appl. Crystallogr. 54, 1006–1011. 10.1107/S1600576721002910 34188619 PMC8202033

[B52] Spek A. (2009). Structure validation in chemical crystallography. Acta Crystallogr. Sect. D. 65, 148–155. 10.1107/S090744490804362X 19171970 PMC2631630

[B53] Tang B. Z. Qin A. (2013). Aggregation-induced emission: Applications. John Wiley & Sons.

[B54] Tauc J. (1968). Optical properties and electronic structure of amorphous Ge and Si. Mater. Res. Bull. 3, 37–46. 10.1016/0025-5408(68)90023-8

[B55] Tiekink E. R. T. (2014). Molecular crystals by design? Chem. Commun. 50, 11079–11082. 10.1039/C4CC04972A 25130670

[B56] Udayakumar M. Ceron M. Ceballos P. Percino J. Thamotharan S. (2019a). Quantitative analysis of weak non-covalent interactions in (Z)-3-(4-chlorophenyl)-2-phenylacrylonitrile: Insights from PIXEL and Hirshfeld surface analysis. Acta Crystallogr. Sect. E 75, 499–505. 10.1107/S2056989019003694 PMC6509683 31161064

[B57] Udayakumar M. Cerón M. Ceballos P. Percino M. J. Thamotharan S. (2019b). Interplay of weak noncovalent interactions in two conjugated positional isomers: A combined X-ray, optical properties and theoretical investigation. J. Mol. Struct. 1195, 32–42. 10.1016/j.molstruc.2019.05.109

[B58] Udayakumar M. Cerón M. Ceballos P. Venkatesan P. Percino M. J. Thamotharan S. (2019c). A quantitative study of weak noncovalent interactions in two pyridine isomers containing nitrile and thiophene moieties: A combined X-ray and theoretical investigation. J. Chem. Sci. 131, 60. 10.1007/s12039-019-1636-3

[B59] Udayakumar M. Cerón M. Ceballos P. Percino M. J. Thamotharan S. (2020). Correlation between structural and optical properties of π-conjugated acrylonitrile derivatives: Insights from X-ray, energy frameworks, TD-DFT and charge density analysis. J. Mol. Struct. 1213, 128174. 10.1016/j.molstruc.2020.128174

[B60] Venkatesan P. Cerón M. Thamotharan S. Robles F. Percino M. J. (2018). Quantitative analysis of weak non-covalent interactions in (Z)-3-(4-halophenyl)-2-(pyridin-2/3/4-yl)acrylonitriles. CrystEngComm 20, 2681–2697. 10.1039/C7CE02096A

[B61] Wang C. Li Z. (2017). Molecular conformation and packing: Their critical roles in the emission performance of mechanochromic fluorescence materials. Mater. Chem. Front. 1, 2174–2194. 10.1039/C7QM00201G

[B62] Wang X. Wolfbeis O. S. Meier R. J. (2013). Luminescent probes and sensors for temperature. Chem. Soc. Rev. 42, 7834–7869. 10.1039/C3CS60102A 23793774

[B63] Wang Y. Shang H. Li B. Jiang S. (2022). Reversible luminescence “off–on” regulation based on tunable photodimerization via crystal-to-cocrystal transformation. J. Mater. Chem. C 10, 734–741. 10.1039/D1TC04401J

[B64] Wu C. Chiu D. T. (2013). Highly fluorescent semiconducting polymer dots for biology and medicine. Angew. Chem. Int. Ed. Engl. 52, 3086–3109. 10.1002/anie.201205133 23307291 PMC5616106

[B65] Wu H. Hang C. Li X. Yin L. Zhu M. Zhang J. (2017). Molecular stacking dependent phosphorescence–fluorescence dual emission in a single luminophore for self-recoverable mechanoconversion of multicolor luminescence. Chem. Commun. 53, 2661–2664. 10.1039/C6CC04901J 27424946

[B66] Yan D. Evans D. G. (2014). Molecular crystalline materials with tunable luminescent properties: From polymorphs to multi-component solids. Mater. Horizons 1, 46–57. 10.1039/C3MH00023K

[B67] Zhan Y. Wei Q. Zhao J. Zhang X. (2017). Reversible mechanofluorochromism and acidochromism using a cyanostyrylbenzimidazole derivative with aggregation-induced emission. RSC Adv. 7, 48777–48784. 10.1039/C7RA09131A

[B68] Zhao Y. Truhlar D. G. (2008). The M06 suite of density functionals for main group thermochemistry, thermochemical kinetics, noncovalent interactions, excited states, and transition elements: Two new functionals and systematic testing of four M06-class functionals and 12 other functionals. Theor. Chem. Acc. 120, 215–241. 10.1007/s00214-007-0310-x

[B69] Zhou Z. Yuan J. Yang R. (2009). Efficient Knoevenagel condensation catalyzed by 2-hydroxyethylammonium acetate under solvent-free conditions at room temperature. Synth. Commun. 39, 2001–2007. 10.1080/00397910802632530

