# Supplementary material for: Weak noncovalent interactions in two positional isomers of acrylonitrile derivatives: inputs from PIXEL energy, Hirshfeld surface and QTAIM analyses
Source: Front Chem. 2023 Jun 28;11:1209428. doi: 10.3389/fchem.2023.1209428 (PMC10338114; doi:10.3389/fchem.2023.1209428)
Supplement: Supplementary file 1 [file DataSheet2.docx]

**Checkcif report: crystal 1**

No syntax errors found. [CIF dictionary](http://www.iucr.org/iucr-top/cif/cif_core/definitions/index.html)
Please wait while processing .... [Interpreting this report](http://journals.iucr.org/services/cif/checking/checkcifreport.html)

**Datablock: shelx**

| Bond precision: | C-C = 0.0015 A | Wavelength=1.54178 |
| --- | --- | --- |

| Cell: | a=9.38435(9) | b=35.1152(3) | c=9.42933(9) |
| --- | --- | --- | --- |
|  | alpha=90 | beta=99.4298(9) | gamma=90 |
| Temperature: | 110 K |  |  |

|  | Calculated | Reported |
| --- | --- | --- |
| Volume | 3065.29(5) | 3065.29(5) |
| Space group | P 21/n | P 21/n |
| Hall group | -P 2yn | -P 2yn |
| Moiety formula | C21 H16 N2 | C21 H16 N2 |
| Sum formula | C21 H16 N2 | C21 H16 N2 |
| Mr | 296.36 | 296.36 |
| Dx,g cm-3 | 1.284 | 1.284 |
| Z | 8 | 8 |
| Mu (mm-1) | 0.586 | 0.586 |
| F000 | 1248.0 | 1248.0 |
| F000' | 1251.31 |  |
| h,k,lmax | 11,43,11 | 11,43,11 |
| Nref | 6021 | 6004 |
| Tmin,Tmax | 0.833,0.895 | 0.861,0.913 |
| Tmin' | 0.810 |  |

| Correction method= # Reported T Limits: Tmin=0.861 Tmax=0.913 AbsCorr = ANALYTICAL |  |
| --- | --- |

| Data completeness= 0.997 | Theta(max)= 71.878 |
| --- | --- |

| R(reflections)= 0.0354( 5432) | wR2(reflections)= 0.0998( 6004) |
| --- | --- |
| \| S = 1.045 \| Npar= 449 \| \| --- \| --- \| |  |

The following ALERTS were generated. Each ALERT has the format

**test-name_ALERT_alert-type_alert-level**.

Click on the hyperlinks for more details of the test.


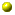
**Alert level C**

[PLAT911_ALERT_3_C](javascript:makeHelpWindow(%22PLAT911.html%22)) Missing FCF Refl Between Thmin & STh/L= 0.600 14 Report

[PLAT913_ALERT_3_C](javascript:makeHelpWindow(%22PLAT913.html%22)) Missing # of Very Strong Reflections in FCF .... 11 Note


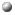
**Alert level G**

[PLAT002_ALERT_2_G](javascript:makeHelpWindow(%22PLAT002.html%22)) Number of Distance or Angle Restraints on AtSite 24 Note

[PLAT142_ALERT_4_G](javascript:makeHelpWindow(%22PLAT142.html%22)) s.u. on b - Axis Small or Missing .............. 0.00030 Ang.

[PLAT143_ALERT_4_G](javascript:makeHelpWindow(%22PLAT143.html%22)) s.u. on c - Axis Small or Missing .............. 0.00009 Ang.

[PLAT168_ALERT_4_G](javascript:makeHelpWindow(%22PLAT168.html%22)) The CIF-Embedded .res File Contains EXYZ Records 2 Report

[PLAT171_ALERT_4_G](javascript:makeHelpWindow(%22PLAT171.html%22)) The CIF-Embedded .res File Contains EADP Records 12 Report

[PLAT174_ALERT_4_G](javascript:makeHelpWindow(%22PLAT174.html%22)) The CIF-Embedded .res File Contains FLAT Records 2 Report

[PLAT175_ALERT_4_G](javascript:makeHelpWindow(%22PLAT175.html%22)) The CIF-Embedded .res File Contains SAME Records 2 Report

[PLAT230_ALERT_2_G](javascript:makeHelpWindow(%22PLAT230.html%22)) Hirshfeld Test Diff for C7A --C15A . 6.6 s.u.

[PLAT230_ALERT_2_G](javascript:makeHelpWindow(%22PLAT230.html%22)) Hirshfeld Test Diff for C20A --C21A . 5.3 s.u.

[PLAT301_ALERT_3_G](javascript:makeHelpWindow(%22PLAT301.html%22)) Main Residue Disorder ..............(Resd 1 ) 26% Note

[PLAT301_ALERT_3_G](javascript:makeHelpWindow(%22PLAT301.html%22)) Main Residue Disorder ..............(Resd 2 ) 26% Note

[PLAT410_ALERT_2_G](javascript:makeHelpWindow(%22PLAT410.html%22)) Short Intra H...H Contact H11A ..H21C . 1.95 Ang.

x,y,z = 1_555 Check

**And 3 other PLAT410 Alerts**

More ...

[PLAT811_ALERT_5_G](javascript:makeHelpWindow(%22PLAT811.html%22)) No ADDSYM Analysis: Too Many Excluded Atoms .... ! Info

[PLAT860_ALERT_3_G](javascript:makeHelpWindow(%22PLAT860.html%22)) Number of Least-Squares Restraints ............. 42 Note

[PLAT883_ALERT_1_G](javascript:makeHelpWindow(%22PLAT883.html%22)) No Info/Value for _atom_sites_solution_primary . Please Do !

[PLAT910_ALERT_3_G](javascript:makeHelpWindow(%22PLAT910.html%22)) Missing # of FCF Reflection(s) Below Theta(Min). 1 Note

[PLAT912_ALERT_4_G](javascript:makeHelpWindow(%22PLAT912.html%22)) Missing # of FCF Reflections Above STh/L= 0.600 2 Note

[PLAT941_ALERT_3_G](javascript:makeHelpWindow(%22PLAT941.html%22)) Average HKL Measurement Multiplicity ........... 3.3 Low

[PLAT967_ALERT_5_G](javascript:makeHelpWindow(%22PLAT967.html%22)) Note: Two-Theta Cutoff Value in Embedded .res .. 143.8 Degree

[PLAT978_ALERT_2_G](javascript:makeHelpWindow(%22PLAT978.html%22)) Number C-C Bonds with Positive Residual Density. 19 Info

0 **ALERT level A** = Most likely a serious problem - resolve or explain

0 **ALERT level B** = A potentially serious problem, consider carefully

2 **ALERT level C** = Check. Ensure it is not caused by an omission or oversight

23 **ALERT level G** = General information/check it is not something unexpected

1 ALERT type 1 CIF construction/syntax error, inconsistent or missing data

8 ALERT type 2 Indicator that the structure model may be wrong or deficient

7 ALERT type 3 Indicator that the structure quality may be low

7 ALERT type 4 Improvement, methodology, query or suggestion

2 ALERT type 5 Informative message, check

**Checkcif report: crystal 2i**

No syntax errors found. [CIF dictionary](http://www.iucr.org/iucr-top/cif/cif_core/definitions/index.html)
Please wait while processing .... [Interpreting this report](http://journals.iucr.org/services/cif/checking/checkcifreport.html)

**Datablock: shelx**

| Bond precision: | C-C = 0.0030 A | Wavelength=0.71073 |
| --- | --- | --- |

| Cell: | a=9.3422(4) | b=11.5706(5) | c=15.1593(7) |
| --- | --- | --- | --- |
|  | alpha=90.219(3) | beta=93.027(4) | gamma=111.520(4) |
| Temperature: | 110 K |  |  |

|  | Calculated | Reported |
| --- | --- | --- |
| Volume | 1521.81(12) | 1521.81(12) |
| Space group | P -1 | P -1 |
| Hall group | -P 1 | -P 1 |
| Moiety formula | C21 H16 N2 | C21 H16 N2 |
| Sum formula | C21 H16 N2 | C21 H16 N2 |
| Mr | 296.36 | 296.36 |
| Dx,g cm-3 | 1.293 | 1.293 |
| Z | 4 | 4 |
| Mu (mm-1) | 0.076 | 0.076 |
| F000 | 624.0 | 624.0 |
| F000' | 624.21 |  |
| h,k,lmax | 11,13,18 | 11,13,18 |
| Nref | 5352 | 5350 |
| Tmin,Tmax | 0.990,0.993 | 0.977,0.995 |
| Tmin' | 0.961 |  |

| Correction method= # Reported T Limits: Tmin=0.977 Tmax=0.995 AbsCorr = ANALYTICAL |  |
| --- | --- |

| Data completeness= 1.000 | Theta(max)= 24.999 |
| --- | --- |

| R(reflections)= 0.0459( 3539) | wR2(reflections)= 0.1194( 5350) |
| --- | --- |
| \| S = 1.025 \| Npar= 416 \| \| --- \| --- \| |  |

The following ALERTS were generated. Each ALERT has the format

**test-name_ALERT_alert-type_alert-level**.

Click on the hyperlinks for more details of the test.


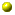
**Alert level C**

[PLAT230_ALERT_2_C](javascript:makeHelpWindow(%22PLAT230.html%22)) Hirshfeld Test Diff for C20B --C21B . 6.9 s.u.

[PLAT906_ALERT_3_C](javascript:makeHelpWindow(%22PLAT906.html%22)) Large K Value in the Analysis of Variance ...... 8.217 Check


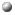
**Alert level G**

[PLAT230_ALERT_2_G](javascript:makeHelpWindow(%22PLAT230.html%22)) Hirshfeld Test Diff for C7A --C15A . 6.1 s.u.

[PLAT300_ALERT_4_G](javascript:makeHelpWindow(%22PLAT300.html%22)) Atom Site Occupancy of H22D Constrained at 0.5 Check

**And 5 other PLAT300 Alerts**

More ...

[PLAT367_ALERT_2_G](javascript:makeHelpWindow(%22PLAT367.html%22)) Long? C(sp?)-C(sp?) Bond C4B - C22B . 1.50 Ang.

[PLAT883_ALERT_1_G](javascript:makeHelpWindow(%22PLAT883.html%22)) No Info/Value for _atom_sites_solution_primary . Please Do !

[PLAT909_ALERT_3_G](javascript:makeHelpWindow(%22PLAT909.html%22)) Percentage of I>2sig(I) Data at Theta(Max) Still 47% Note

[PLAT910_ALERT_3_G](javascript:makeHelpWindow(%22PLAT910.html%22)) Missing # of FCF Reflection(s) Below Theta(Min). 2 Note

[PLAT933_ALERT_2_G](javascript:makeHelpWindow(%22PLAT933.html%22)) Number of HKL-OMIT Records in Embedded .res File 1 Note

[PLAT941_ALERT_3_G](javascript:makeHelpWindow(%22PLAT941.html%22)) Average HKL Measurement Multiplicity ........... 3.1 Low

[PLAT967_ALERT_5_G](javascript:makeHelpWindow(%22PLAT967.html%22)) Note: Two-Theta Cutoff Value in Embedded .res .. 50.0 Degree

[PLAT978_ALERT_2_G](javascript:makeHelpWindow(%22PLAT978.html%22)) Number C-C Bonds with Positive Residual Density. 7 Info

0 **ALERT level A** = Most likely a serious problem - resolve or explain

0 **ALERT level B** = A potentially serious problem, consider carefully

2 **ALERT level C** = Check. Ensure it is not caused by an omission or oversight

15 **ALERT level G** = General information/check it is not something unexpected

1 ALERT type 1 CIF construction/syntax error, inconsistent or missing data

5 ALERT type 2 Indicator that the structure model may be wrong or deficient

4 ALERT type 3 Indicator that the structure quality may be low

6 ALERT type 4 Improvement, methodology, query or suggestion

1 ALERT type 5 Informative message, check

**Checkcif report: crystal 2j**

No syntax errors found. [CIF dictionary](http://www.iucr.org/iucr-top/cif/cif_core/definitions/index.html)
Please wait while processing .... [Interpreting this report](http://journals.iucr.org/services/cif/checking/checkcifreport.html)

**Datablock: shelx**

| Bond precision: | C-C = 0.0030 A | Wavelength=1.54178 |
| --- | --- | --- |

| Cell: | a=9.3470(5) | b=11.5742(8) | c=15.1532(7) |
| --- | --- | --- | --- |
|  | alpha=90.225(4) | beta=93.038(4) | gamma=111.568(6) |
| Temperature: | 110 K |  |  |

|  | Calculated | Reported |
| --- | --- | --- |
| Volume | 1521.93(16) | 1521.92(16) |
| Space group | P -1 | P -1 |
| Hall group | -P 1 | -P 1 |
| Moiety formula | C21 H16 N2 | C21 H16 N2 |
| Sum formula | C21 H16 N2 | C21 H16 N2 |
| Mr | 296.36 | 296.36 |
| Dx,g cm-3 | 1.293 | 1.293 |
| Z | 4 | 4 |
| Mu (mm-1) | 0.590 | 0.590 |
| F000 | 624.0 | 624.0 |
| F000' | 625.66 |  |
| h,k,lmax | 11,14,18 | 11,14,18 |
| Nref | 5982 | 5973 |
| Tmin,Tmax | 0.965,0.982 | 0.964,0.989 |
| Tmin' | 0.948 |  |

| Correction method= # Reported T Limits: Tmin=0.964 Tmax=0.989 AbsCorr = ANALYTICAL |  |
| --- | --- |

| Data completeness= 0.998 | Theta(max)= 71.894 |
| --- | --- |

| R(reflections)= 0.0449( 3647) | wR2(reflections)= 0.1209( 5973) |
| --- | --- |
| \| S = 0.982 \| Npar= 417 \| \| --- \| --- \| |  |

The following ALERTS were generated. Each ALERT has the format

**test-name_ALERT_alert-type_alert-level**.

Click on the hyperlinks for more details of the test.


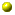
**Alert level C**

[PLAT906_ALERT_3_C](javascript:makeHelpWindow(%22PLAT906.html%22)) Large K Value in the Analysis of Variance ...... 2.275 Check

[PLAT911_ALERT_3_C](javascript:makeHelpWindow(%22PLAT911.html%22)) Missing FCF Refl Between Thmin & STh/L= 0.600 2 Report


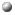
**Alert level G**

[PLAT066_ALERT_1_G](javascript:makeHelpWindow(%22PLAT066.html%22)) Predicted and Reported Tmin&Tmax Range Identical ? Check

[PLAT883_ALERT_1_G](javascript:makeHelpWindow(%22PLAT883.html%22)) No Info/Value for _atom_sites_solution_primary . Please Do !

[PLAT912_ALERT_4_G](javascript:makeHelpWindow(%22PLAT912.html%22)) Missing # of FCF Reflections Above STh/L= 0.600 7 Note

[PLAT941_ALERT_3_G](javascript:makeHelpWindow(%22PLAT941.html%22)) Average HKL Measurement Multiplicity ........... 3.0 Low

[PLAT967_ALERT_5_G](javascript:makeHelpWindow(%22PLAT967.html%22)) Note: Two-Theta Cutoff Value in Embedded .res .. 143.8 Degree

[PLAT978_ALERT_2_G](javascript:makeHelpWindow(%22PLAT978.html%22)) Number C-C Bonds with Positive Residual Density. 16 Info

0 **ALERT level A** = Most likely a serious problem - resolve or explain

0 **ALERT level B** = A potentially serious problem, consider carefully

2 **ALERT level C** = Check. Ensure it is not caused by an omission or oversight

6 **ALERT level G** = General information/check it is not something unexpected

2 ALERT type 1 CIF construction/syntax error, inconsistent or missing data

1 ALERT type 2 Indicator that the structure model may be wrong or deficient

3 ALERT type 3 Indicator that the structure quality may be low

1 ALERT type 4 Improvement, methodology, query or suggestion

1 ALERT type 5 Informative message, check
