# Supplementary material for: Weak noncovalent interactions in two positional isomers of acrylonitrile derivatives: inputs from PIXEL energy, Hirshfeld surface and QTAIM analyses
Source: Front Chem. 2023 Jun 28;11:1209428. doi: 10.3389/fchem.2023.1209428 (PMC10338114; doi:10.3389/fchem.2023.1209428)
Supplement: Supplementary file 2 [file DataSheet1.docx]

Weak Noncovalent Interactions in Two Positional Isomers of Acrylonitrile Derivatives: Inputs from PIXEL Energy, Hirshfeld Surface and QTAIM Analyses

M. Judith Percino^1*^, Mani Udayakumar^2^, Margarita Cerón^1^, Enrique Pérez-Gutiérrez^1^, Perumal Venkatesan^3^, Subbiah Thamotharan^2,^*

^1^Unidad de Polímeros y Electrónica Orgánica, Instituto de Ciencias, Benemérita Universidad Autónoma de Puebla, Val3-Ecocampus Valsequillo, Independencia O2 Sur 50, San Pedro Zacachimalpa, Puebla 72960, CP, México

^2^Biomolecular Crystallography Laboratory, Department of Bioinformatics, School of Chemical and Biotechnology, SASTRA Deemed University, Thanjavur 613 401, India

^3^Department of Chemistry, Srimad Andavan Arts and Science College (Autonomous), Tiruchirappalli 620 005, India

*** Correspondence:**Corresponding Authors
Subbiah Thamotharan, [thamu@scbt.sastra.edu](mailto:thamu@scbt.sastra.edu), M. Judith Percino, [judith.percino@correo.buap.mx](mailto:judith.percino@correo.buap.mx)


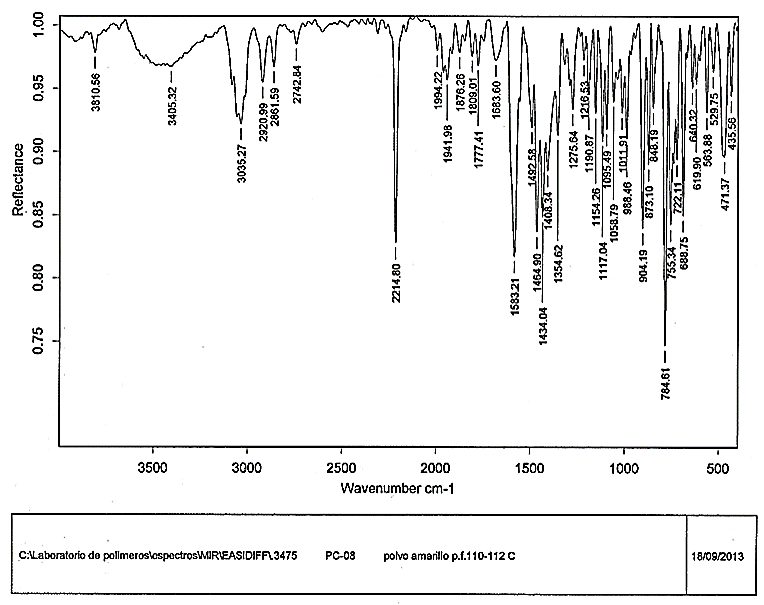


**Figure S1**. FT-IR spectrum of compound **1**.

**Figure S2**. FT-IR spectrum of compound **2**.

**Figure S3**. ^1^H and ^13^C NMR spectra of **1** in CDCl_3_.

**Figure S4**. ^1^H and ^13^C NMR spectra of **2** in CDCl_3_.


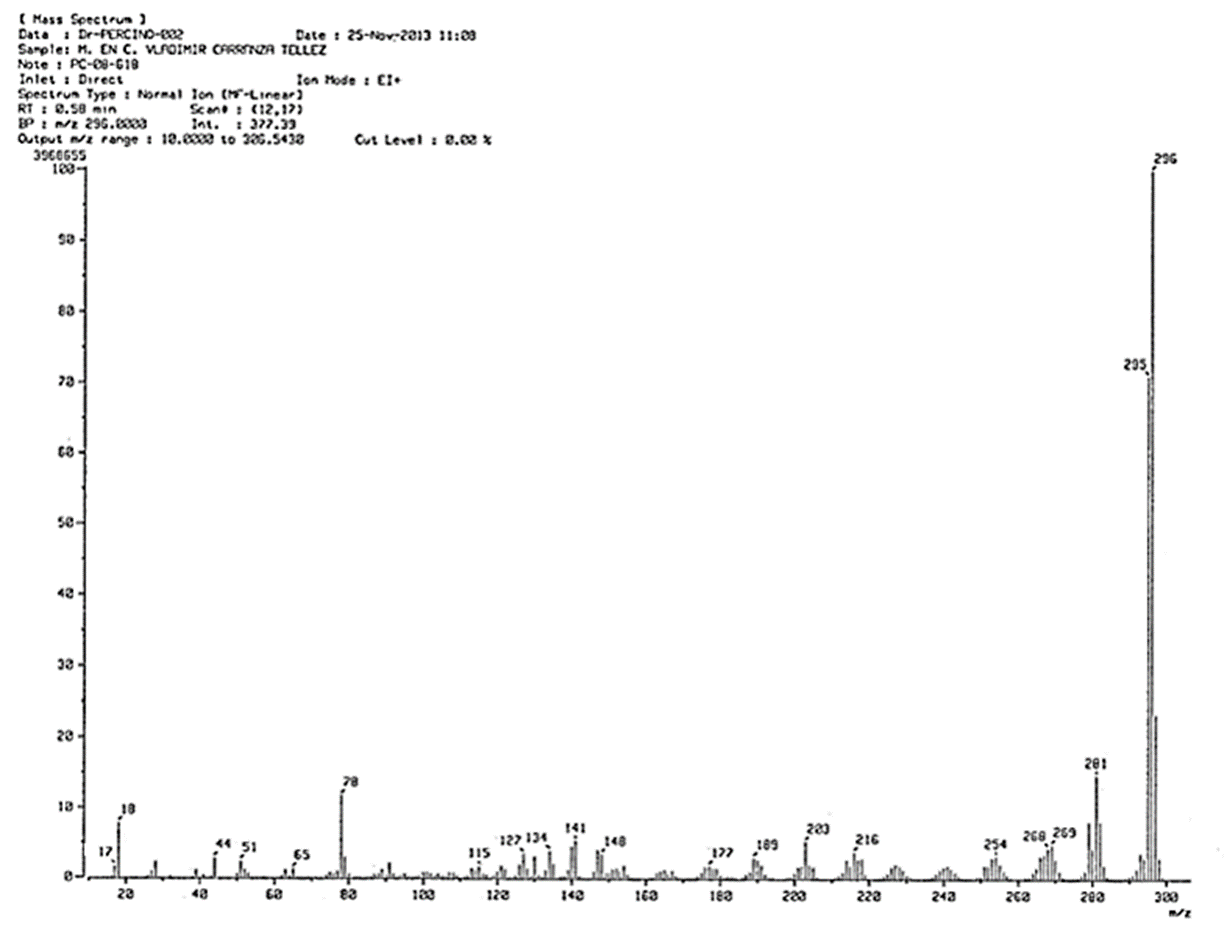


**Figure S5**. EI mass spectrum for compound **1**.


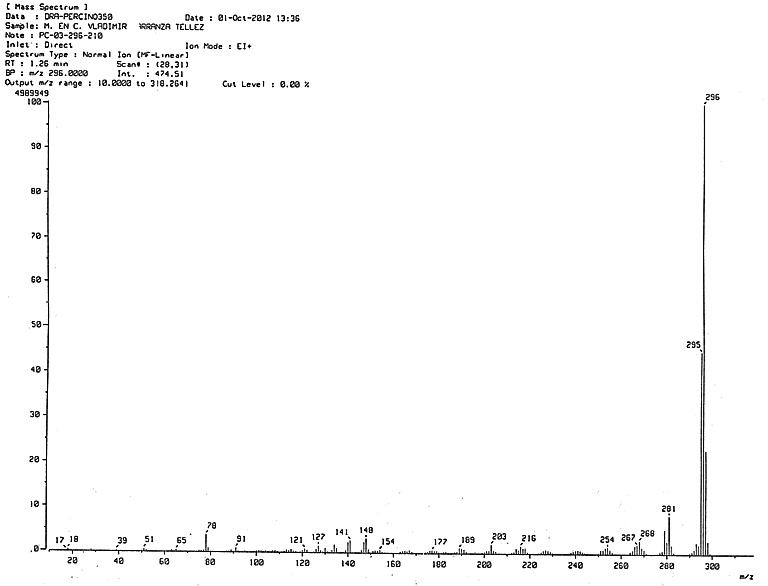


**Figure S6**. EI mass spectrum for compound **2**.

**Table S1**. Dihedral angle (°) is formed between different groups in **1**.

|  | Ring B | Ring C | Acrylonitrile^a^ |
| --- | --- | --- | --- |
| Molecule A (major disordered component) | | | |
| Ring A | 0.68 | 11.41 | 27.58 |
| Ring B |  | 10.95 | 27.33 |
| Ring C |  |  | 17.51 |
| Molecule B (major disordered component) | | | |
| Ring A | 0.78 | 13.48 | 21.87 |
| Ring B |  | 14.07 | 22.60 |
| Ring C |  |  | 10.10 |

^a –^ Acrylonitrile plane is defined by atoms C1A/C7A/C8A/C15A/N2A (Molecule A) and C1B/C7B/C8B/C15B/N2B (Molecule B).

**Table S2**. Dihedral angle (°) is formed between different groups in **2j** (values in parenthesis correspond to the molecules of **2i**).

|  | Ring B | Ring C | Acrylonitrile^a^ |
| --- | --- | --- | --- |
| Molecule A | | | |
| Ring A | 4.97 (4.90) | 2.23 (2.46) | 24.35 (24.34) |
| Ring B |  | 5.90 (5.87) | 29.30 (29.22) |
| Ring C |  |  | 24.23 (24.24) |
| Molecule B | | | |
| Ring A | 3.53 (3.63) | 6.65 (6.78) | 24.72 (24.81) |
| Ring B |  | 6.30 (7.39) | 28.24 (28.43) |
| Ring C |  |  | 25.57 (25.79) |

^a –^ Acrylonitrile plane is defined by atoms C1A/C7A/C8A/C15A/N2A (Molecule A) and C1B/C7B/C8B/C15B/N2B (Molecule B).

**Table S3**. Comparison of bond lengths between X-ray and optimized structures of **1** (molecules A and B).

| X-ray | | Optimized | | Difference |
| --- | --- | --- | --- | --- |
| Bond | Length (Å) | Bond | Length (Å) |  |
| N3A-C18A | 1.331 | N1-C2 | 1.326 | 0.005 |
| N3A-C16A | 1.323 | N1-C10 | 1.336 | -0.013 |
| C18A-C19A | 1.383 | C2-C4 | 1.387 | -0.004 |
| C19A-C20A | 1.403 | C4-C6 | 1.385 | 0.018 |
| C20A-C21A | 1.402 | C6-C8 | 1.383 | 0.019 |
| C21A-C16A | 1.438 | C8-C10 | 1.395 | 0.043 |
| C16A-C12A | 1.4896 | C10-C11 | 1.484 | 0.0056 |
| C12A-C11A | 1.3989 | C11-C12 | 1.394 | 0.0049 |
| C12A-C13A | 1.4013 | C11-C19 | 1.395 | 0.0063 |
| C11A-C10A | 1.3843 | C12-C14 | 1.38 | 0.0043 |
| C10A-C9A | 1.4008 | C14-C16 | 1.4 | 0.0008 |
| C9A-C14A | 1.4006 | C16-C17 | 1.398 | 0.0026 |
| C9A-C8A | 1.4638 | C16-C21 | 1.46 | 0.0038 |
| C14A-C13A | 1.3861 | C17-C19 | 1.382 | 0.0041 |
| C8A-C7A | 1.3477 | C21-C23 | 1.345 | 0.0027 |
| C7A-C15A | 1.4422 | C23-C24 | 1.435 | 0.0072 |
| C7A-C1A | 1.489 | C23-C25 | 1.483 | 0.006 |
| C15A-N2A | 1.1493 | C24-N39 | 1.149 | 0.0003 |
| C1A-C2A | 1.4008 | C25-C26 | 1.395 | 0.0058 |
| C1A-C6A | 1.3935 | C25-C33 | 1.392 | 0.0015 |
| C2A-C3A | 1.3817 | C26-C28 | 1.383 | -0.0013 |
| C3A-C4A | 1.3866 | C28-C30 | 1.388 | -0.0014 |
| C4A-C5A | 1.3906 | C30-C32 | 1.39 | 0.0006 |
| C5A-C6A | 1.3983 | C32-C33 | 1.391 | 0.0073 |
| C5A-C22A | 1.5028 | C32-C35 | 1.503 | -0.0002 |
| N3B-C18B | 1.3383 | N1-C2 | 1.326 | 0.0123 |
| N3B-C16B | 1.3467 | N1-C10 | 1.336 | 0.0107 |
| C18B-C19B | 1.385 | C2-C4 | 1.387 | -0.002 |
| C19B-C20B | 1.3847 | C4-C6 | 1.385 | -0.0003 |
| C20B-C21B | 1.391 | C6-C8 | 1.383 | 0.008 |
| C21B-C16B | 1.396 | C8-C10 | 1.396 | 0 |
| C16B-C12B | 1.4896 | C10-C11 | 1.483 | 0.0066 |
| C12B-C11B | 1.3981 | C11-C12 | 1.394 | 0.0041 |
| C12B-C13B | 1.4014 | C11-C19 | 1.395 | 0.0064 |
| C11B-C10B | 1.3836 | C12-C14 | 1.38 | 0.0036 |
| C10B-C9B | 1.401 | C14-C16 | 1.4 | 0.001 |
| C9B-C14B | 1.4023 | C16-C17 | 1.399 | 0.0033 |
| C9B-C8B | 1.4643 | C16-C21 | 1.459 | 0.0053 |
| C14B-C13B | 1.3877 | C17-C19 | 1.382 | 0.0057 |
| C8B-C7B | 1.3499 | C21-C23 | 1.346 | 0.0039 |
| C7B-C15B | 1.4437 | C23-C24 | 1.435 | 0.0087 |
| C7B-C1B | 1.4873 | C23-C25 | 1.484 | 0.0033 |
| C15B-N2B | 1.1491 | C24-N39 | 1.149 | 1E-04 |
| C1B-C2B | 1.3978 | C25-C26 | 1.395 | 0.0028 |
| C1B-C6B | 1.3974 | C25-C33 | 1.393 | 0.0044 |
| C2B-C3B | 1.3827 | C26-C28 | 1.384 | -0.0013 |
| C3B-C4B | 1.3879 | C28-C30 | 1.387 | 0.0009 |
| C4B-C5B | 1.3893 | C30-C32 | 1.391 | -0.0017 |
| C5B-C6B | 1.3957 | C32-C33 | 1.39 | 0.0057 |
| C5B-C22B | 1.5057 | C32-C35 | 1.503 | 0.0027 |

**Table S4**. Comparison of bond angles between X-ray and optimized structures of **1** (molecules A and B).

| X-ray | | Optimized | | Difference |
| --- | --- | --- | --- | --- |
| Atoms | Angle (°) | Atoms | Angle (°) |  |
| C16A-N3A-C18A | 119.6 | C10-N1-C2 | 118.6 | 1 |
| N3A-C18A-C19A | 124.7 | N1-C2-C4 | 123.6 | 1.1 |
| C18A-C19A-C20A | 117.1 | C2-C4-C6 | 117.9 | -0.8 |
| C21A-C20A-C19A | 119.4 | C6-C8-C4 | 119 | 0.4 |
| C20A-C21A-C16A | 118.3 | C6-C8-C10 | 119.1 | -0.8 |
| N3A-C16A-C21A | 120.9 | N1-C10-C8 | 121.7 | -0.8 |
| N3A-C16A-C12A | 117.5 | N1-C10-C11 | 116.5 | 1 |
| C21A-C16A-C12A | 121.6 | C8-C10-C11 | 121.8 | -0.2 |
| C11A-C12A-C16A | 120.54 | C12-C11-C10 | 119.7 | 0.84 |
| C11A-C12A-C13A | 117.82 | C12-C11-C19 | 118.4 | -0.58 |
| C13A-C12A-C16A | 121.62 | C11-C19-C10 | 121.9 | -0.28 |
| C10A-C11A-C12A | 120.88 | C14-C12-C11 | 120.4 | 0.48 |
| C11A-C10A-C9A | 121.34 | C12-C14-C16 | 121.5 | -0.16 |
| C10A-C9A-C8A | 117.52 | C14-C16-C21 | 117.1 | 0.42 |
| C14A-C9A-C10A | 117.87 | C17-C16-C14 | 117.9 | -0.03 |
| C14A-C9A-C8A | 124.55 | C17-C16-C21 | 124.9 | -0.35 |
| C13A-C14A-C9A | 120.72 | C19-C17-C16 | 120.5 | 0.22 |
| C14A-C13A-C12A | 121.36 | C17-C19-C11 | 121.3 | 0.06 |
| C7A-C8A-C9A | 130.68 | C23-C21-C16 | 130.9 | -0.22 |
| C8A-C7A-C15A | 121.13 | C21-C23-C24 | 121.9 | -0.77 |
| C8A-C7A-C1A | 123.89 | C21-C23-C25 | 122.8 | 1.09 |
| C15A-C7A-C1A | 114.96 | C24-C23-C25 | 115.3 | -0.34 |
| N2A-C15A-C7A | 176.53 | N39-C24-C23 | 177.3 | -0.77 |
| C2A-C1A-C7A | 121.09 | C26-C25-C23 | 120.8 | 0.29 |
| C6A-C1A-C7A | 120.42 | C23-C25-C33 | 120.2 | 0.22 |
| C6A-C1A-C2A | 118.48 | C33-C25-C26 | 119 | -0.52 |
| C3A-C2A-C1A | 120.1 | C28-C26-C25 | 120 | 0.1 |
| C2A-C3A-C4A | 120.84 | C26-C28-C30 | 120.4 | 0.44 |
| C3A-C4A-C5A | 120.34 | C28-C30-C32 | 120.6 | -0.26 |
| C4A-C5A-C6A | 118.5 | C30-C32-C33 | 118.5 | 0 |
| C4A-C5A-C22A | 121.02 | C30-C32-C35 | 121.1 | -0.08 |
| C6A-C5A-C22A | 120.47 | C33-C32-C35 | 120.3 | 0.17 |
| C1A-C6A-C5A | 121.73 | C25-C33-C32 | 121.5 | 0.23 |
| C18B-N3B-C16B | 117.74 | C2-N1-C10 | 118.6 | -0.86 |
| N3B-C18B-C19B | 124.14 | N1-C2-C4 | 123.7 | 0.44 |
| C20B-C19B-C18B | 117.95 | C6-C4-C2 | 117.9 | 0.05 |
| C19B-C20B-C21B | 119.05 | C4-C6-C8 | 119 | 0.05 |
| C20B-C21B-C16B | 119.13 | C6-C8-C10 | 119.1 | 0.03 |
| N3B-C16B-C21B | 121.99 | N1-C10-C8 | 121.7 | 0.29 |
| N3B-C16B-C12B | 116.3 | N1-C10-C11 | 116.5 | -0.2 |
| C21B-C16B-C12B | 121.71 | C8-C10-C11 | 121.7 | 0.01 |
| C11B-C12B-C16B | 119.72 | C12-C11-C10 | 119.8 | -0.08 |
| C11B-C12B-C13B | 117.77 | C12-C11-C19 | 118.4 | -0.63 |
| C13B-C12B-C16B | 122.51 | C19-C11-C10 | 121.8 | 0.71 |
| C10B-C11B-C12B | 120.73 | C14-C12-C11 | 120.3 | 0.43 |
| C11B-C10B-C9B | 121.68 | C12-C14-C16 | 121.6 | 0.08 |
| C10B-C9B-C14B | 117.67 | C14-C16-C17 | 117.8 | -0.13 |
| C10B-C9B-C8B | 116.33 | C14-C16-C21 | 116.9 | -0.57 |
| C14B-C9B-C8B | 125.96 | C17-C16-C21 | 125.3 | 0.66 |
| C13B-C14B-C9B | 120.54 | C19-C17-C16 | 120.5 | 0.04 |
| C14B-C13B-C12B | 121.58 | C17-C19-C11 | 121.4 | 0.18 |
| C7B-C8B-C9B | 131.13 | C23-C21-C16 | 131.5 | -0.37 |
| C8B-C7B-C15B | 120.24 | C21-C23-C24 | 122.1 | -1.86 |
| C8B-C7B-C1B | 123.83 | C21-C23-C25 | 122.8 | 1.03 |
| C15B-C7B-C1B | 115.79 | C24-C23-C25 | 115.1 | 0.69 |
| N2B-C15B-C7B | 177.63 | N39-C24-C23 | 177.4 | 0.23 |
| C2B-C1B-C7B | 121.26 | C26-C25-C23 | 121 | 0.26 |
| C6B-C1B-C7B | 120.54 | C33-C25-C21 | 120.1 | 0.44 |
| C6B-C1B-C2B | 118.12 | C33-C25-C26 | 118.9 | -0.78 |
| C3B-C2B-C1B | 120.33 | C28-C26-C25 | 120 | 0.33 |
| C2B-C3B-C4B | 120.84 | C26-C28-C30 | 120.4 | 0.44 |
| C3B-C4B-C5B | 120.15 | C28-C30-C32 | 120.6 | -0.45 |
| C4B-C5B-C6B | 118.62 | C30-C32-C33 | 118.5 | 0.12 |
| C4B-C5B-C22B | 120.85 | C30-C32-C35 | 121 | -0.15 |
| C6B-C5B-C22B | 120.49 | C33-C32-C35 | 120.5 | -0.01 |
| C5B-C6B-C1B | 121.92 | C32-C33-C25 | 121.5 | 0.42 |

**Table S5**. Comparison of bond lengths between X-ray and optimized structures of **2j** (molecules A and B).

| X-ray | | Optimized | | Difference |
| --- | --- | --- | --- | --- |
| Bond | Length (Å) | Bond | Length (Å) |  |
| C22A-C4A | 1.502 | C1-C5 | 1. 502 | 0 |
| C4A-C5A | 1.399 | C5-C6 | 1.392 | 0.007 |
| C4A-C3A | 1.39 | C5-C13 | 1.392 | -0.002 |
| C5A-C6A | 1.385 | C6-C8 | 1.384 | 0.001 |
| C6A-C1A | 1.393 | C8-C10 | 1.394 | -0.001 |
| C1A-C2A | 1.403 | C10-C11 | 1.394 | 0.009 |
| C1A-C7A | 1.484 | C10-C15 | 1.482 | 0.002 |
| C2A-C3A | 1.394 | C11-C13 | 1.384 | 0.01 |
| C7A-C15A | 1.444 | C15-C16 | 1.435 | 0.009 |
| C7A-C8A | 1.349 | C15-C17 | 1.346 | 0.003 |
| C15A-N3A | 1.149 | C16-N38 | 1.149 | 0 |
| C8A-C9A | 1.466 | C17-C19 | 1.46 | 0.006 |
| C9A-C14A | 1.398 | C19-C20 | 1.398 | 0 |
| C9A-C10A | 1.401 | C19-C27 | 1.4 | 0.001 |
| C14A-C13A | 1.383 | C20-C22 | 1.382 | 0.001 |
| C13A-C12A | 1.401 | C22-C24 | 1.395 | 0.006 |
| C12A-C11A | 1.393 | C24-C25 | 1.394 | -0.001 |
| C12A-C16A | 1.495 | C24-C29 | 1.483 | 0.012 |
| C11A-C10A | 1.386 | C25-C27 | 1.381 | 0.005 |
| C16A-C21A | 1.388 | C29-C30 | 1.396 | -0.008 |
| C16A-N2A | 1.348 | C29-N39 | 1.336 | 0.012 |
| C21A-C20A | 1.382 | C30-C32 | 1.383 | -0.001 |
| C20A-C19A | 1.384 | C32-C34 | 1.385 | -0.001 |
| C19A-C18A | 1.382 | C34-C36 | 1.387 | -0.005 |
| C18A-N2A | 1.345 | C36-N39 | 1.327 | 0.018 |
| C22B-C4B | 1.506 | C1-C5 | 1.502 | 0.004 |
| C4B-C5B | 1.391 | C5-C6 | 1.39 | 0.001 |
| C4B-C3B | 1.401 | C5-C13 | 1.394 | 0.007 |
| C5B-C6B | 1.389 | C6-C8 | 1.386 | 0.003 |
| C6B-C1B | 1.389 | C8-C10 | 1.393 | -0.004 |
| C1B-C2B | 1.398 | C10-C11 | 1.396 | 0.002 |
| C1B-C7B | 1.486 | C10-C15 | 1.482 | 0.004 |
| C2B-C3B | 1.381 | C11-C13 | 1.383 | -0.002 |
| C7B-C15B | 1.447 | C15-C16 | 1.435 | 0.012 |
| C7B-C8B | 1.348 | C15-C17 | 1.346 | 0.002 |
| C15B-N3B | 1.149 | C16-N38 | 1.149 | 0 |
| C8B-C9B | 1.464 | C17-C19 | 1.459 | 0.005 |
| C9B-C14B | 1.4 | C19-C20 | 1.399 | 0.001 |
| C9B-C10B | 1.4 | C19-C27 | 1.4 | 0 |
| C14B-C13B | 1.384 | C20-C22 | 1.382 | 0.002 |
| C13B-C12B | 1.397 | C22-C24 | 1.395 | 0.002 |
| C12B-C11B | 1.397 | C24-C25 | 1.394 | 0.003 |
| C12B-C16B | 1.49 | C24-C29 | 1.483 | 0.007 |
| C11B-C10B | 1.379 | C25-C27 | 1.38 | -0.001 |
| C16B-C21B | 1.387 | C29-C30 | 1.396 | -0.009 |
| C16B-N2B | 1.356 | C29-N39 | 1.336 | 0.02 |
| C21B-C20B | 1.384 | C30-C32 | 1.383 | 0.001 |
| C20B-C19B | 1.375 | C32-C34 | 1.385 | -0.01 |
| C19B-C18B | 1.384 | C34-C36 | 1.387 | -0.003 |
| C18B-N2B | 1.344 | C36-N39 | 1.327 | .004 |

**Table S6**. Comparison of bond angles between X-ray and optimized structures of **2j** (molecules A and B).

| X-ray | | Optimized | | Difference |
| --- | --- | --- | --- | --- |
| Atoms | Angle (°) | Atoms | Angle (°) |  |
| C5A-C4A-C22A | 120.49 | C6-C5-C1 | 121 | -0.51 |
| C3A-C4A-C22A | 121.65 | C13-C5-C1 | 121.1 | 0.55 |
| C3A-C4A-C5A | 117.87 | C13-C5-C6 | 117.9 | -0.03 |
| C6A-C5A-C4A | 121.05 | C8-C6-C5 | 121.2 | -0.15 |
| C5A-C6A-C1A | 121.07 | C6-C8-C10 | 120.7 | 0.37 |
| C6A-C1A-C2A | 118.32 | C8-C10-C11 | 118.3 | 0.02 |
| C6A-C1A-C7A | 120.99 | C8-C10-C15 | 120.5 | 0.49 |
| C2A-C1A-C7A | 120.69 | C13-C10-C15 | 121.2 | -0.51 |
| C3A-C2A-C1A | 120.14 | C5-C13-C10 | 120.7 | -0.56 |
| C4A-C3A-C2A | 121.53 | C1-C5-C13 | 121.2 | 0.33 |
| C15A-C7A-C1A | 114.69 | C16-C15-C10 | 115 | -0.31 |
| C8A-C7A-C1A | 124.46 | C17-C15-C10 | 123.1 | 1.36 |
| C8A-C7A-C15A | 120.84 | C17-C15-C16 | 121.9 | -1.06 |
| N3A-C15A-C7A | 176.8 | N38-C16-C15 | 177.1 | -0.3 |
| C7A-C8A-C9A | 129.72 | C15-C17-C19 | 130.7 | -0.98 |
| C14A-C9A-C8A | 125.12 | C20-C19-C17 | 124.7 | 0.42 |
| C14A-C9A-C10A | 117.6 | C20-C19-C27 | 117.9 | -0.3 |
| C10A-C9A-C8A | 117.28 | C27-C19-C17 | 117.3 | -0.02 |
| C13A-C14A-C9A | 120.99 | C22-C20-C19 | 120.5 | 0.49 |
| C14A-C13A-C12A | 121.27 | C20-C22-C24 | 121.3 | -0.03 |
| C13A-C12A-C16A | 122.29 | C22-C24-C29 | 121.7 | 0.59 |
| C11A-C12A-C13A | 117.89 | C25-C24-C22 | 118.4 | -0.51 |
| C11A-C12A-C16A | 119.81 | C27-C24-C29 | 119.9 | -0.09 |
| C10A-C11A-C12A | 120.86 | C19-C27-C24 | 120.4 | 0.46 |
| C11A-C10A-C9A | 121.39 | C27-C19-C17 | 121.5 | -0.11 |
| C21A-C16A-C12A | 121.73 | C30-C29-C24 | 121.5 | 0.23 |
| N2A-C16A-C12A | 116.23 | N39-C29-C24 | 116.8 | -0.57 |
| N2A-C16A-C21A | 122.04 | N39-C29-C30 | 121.7 | 0.34 |
| C20A-C21A-C16A | 119.31 | C32-C30-C29 | 119.2 | 0.11 |
| C21A-C20A-C19A | 119.16 | C30-C32-C34 | 119 | 0.16 |
| C18A-C19A-C20A | 118.17 | C36-C34-C32 | 117.9 | 0.27 |
| N2A-C18A-C19A | 123.6 | N39-C36-C34 | 123.7 | -0.1 |
| C18A-N2A-C16A | 117.73 | C36-N39-C29 | 118.5 | -0.77 |
| C5B-C4B-C22B | 121.43 | C6-C5-C1 | 121.3 | 0.13 |
| C5B-C4B-C3B | 117.84 | C6-C5-C13 | 117.9 | -0.06 |
| C3B-C4B-C22B | 120.74 | C13-C5-C1 | 120.8 | -0.06 |
| C6B-C5B-C4B | 120.75 | C8-C6-C5 | 121.2 | -0.45 |
| C5B-C6B-C1B | 121.28 | C6-C8-C10 | 120.6 | 0.68 |
| C6B-C1B-C2B | 118.19 | C8-C10-C11 | 118.3 | -0.11 |
| C6B-C1B-C7B | 120.6 | C8-C10-C15 | 120.6 | 0 |
| C2B-C1B-C7B | 121.21 | C11-C10-C15 | 121.2 | 0.01 |
| C3B-C2B-C1B | 120.51 | C13-C11-C10 | 120.7 | -0.19 |
| C2B-C3B-C4B | 121.42 | C11-C13-C5 | 121.2 | 0.22 |
| C15B-C7B-C1B | 114.84 | C16-C15-C10 | 115.1 | -0.26 |
| C8B-C7B-C1B | 124.44 | C17-C15-C10 | 122.8 | 1.64 |
| C8B-C7B-C15B | 120.71 | C17-C15-C16 | 122.1 | -1.39 |
| N3B-C15B-C7B | 176.9 | N38-C16-C15 | 177.3 | -0.4 |
| C7B-C8B-C9B | 129.77 | C15-C17-C19 | 131.6 | -1.83 |
| C14B-C9B-C8B | 124.55 | C20-C19-C17 | 125.4 | -0.85 |
| C14B-C9B-C10B | 117.77 | C20-C19-C27 | 117.8 | -0.03 |
| C10B-C9B-C8B | 117.56 | C27-C19-C17 | 116.7 | 0.86 |
| C13B-C14B-C9B | 120.39 | C22-C20-C19 | 120.4 | -0.01 |
| C14B-C13B-C12B | 121.76 | C20-C22-C24 | 121.4 | 0.36 |
| C13B-C12B-C16B | 122.09 | C22-C24-C29 | 121.8 | 0.29 |
| C11B-C12B-C13B | 117.7 | C25-C24-C22 | 118.4 | -0.7 |
| C11B-C12B-C16B | 120.2 | C25-C24-C29 | 119.8 | 0.4 |
| C10B-C11B-C12B | 120.76 | C27-C25-C24 | 120.3 | 0.46 |
| C11B-C10B-C9B | 121.59 | C25-C27-C19 | 121.6 | -0.01 |
| C21B-C16B-C12B | 121.52 | C30-C29-C24 | 121.7 | -0.18 |
| N2B-C16B-C12B | 116.6 | N39-C29-C30 | 116.6 | 0 |
| N2B-C16B-C21B | 121.87 | N39-C29-C24 | 121.7 | 0.17 |
| C20B-C21B-C16B | 118.9 | C32-C30-C29 | 119.1 | -0.2 |
| C19B-C20B-C21B | 119.95 | C34-C32-C30 | 119 | 0.95 |
| C20B-C19B-C18B | 117.96 | C32-C34-C36 | 117.9 | 0.06 |
| N2B-C18B-C19B | 123.5 | N39-C36-C34 | 123.6 | -0.1 |
| C18B-N2B-C16B | 117.8 | C36-N39-C29 | 118.6 | -0.8 |

**
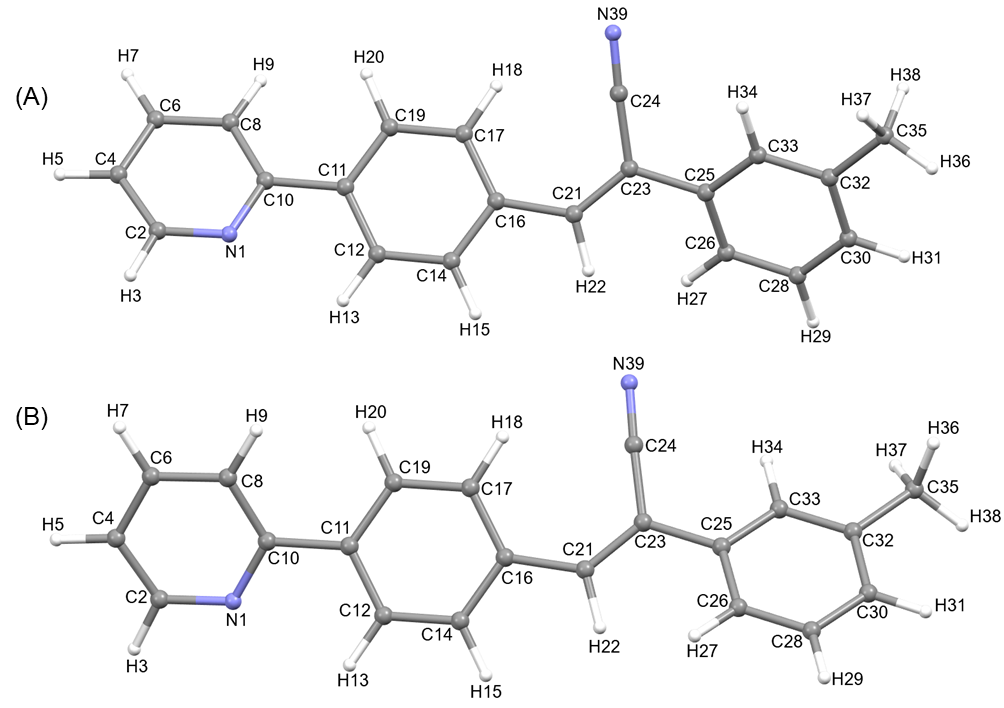
**

**Figure S7.** The optimized structures of **1 (A)** molecule A and **(B)** molecule B.


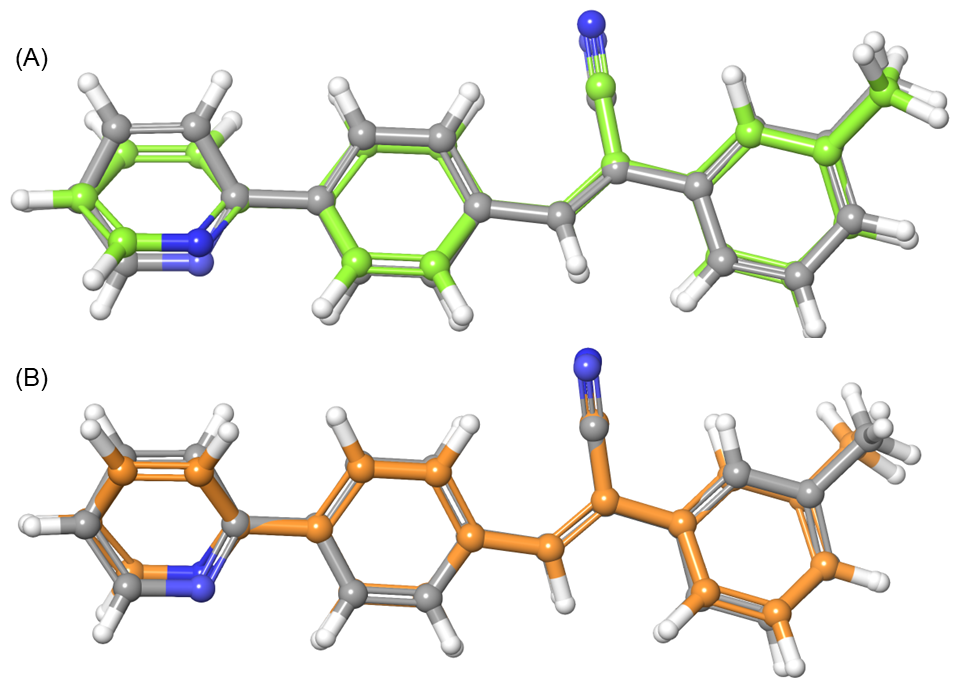


**Figure S8**. Structural overlay between X-ray and optimized structures of **1** (A) molecule A and (B) molecule B. colour codes: grey (X-ray), green (optimized molecule A) and orange (optimized molecule B.

**
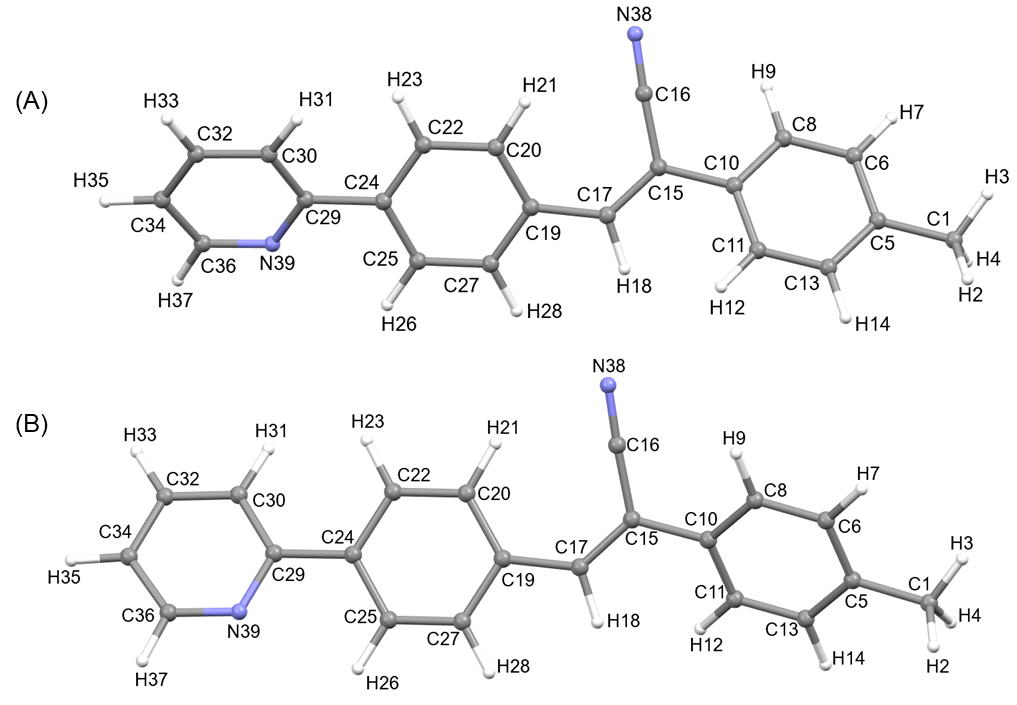
**

**Figure S9**. The optimized structures of **2j (A)** molecule A and **(B)** molecule B

**
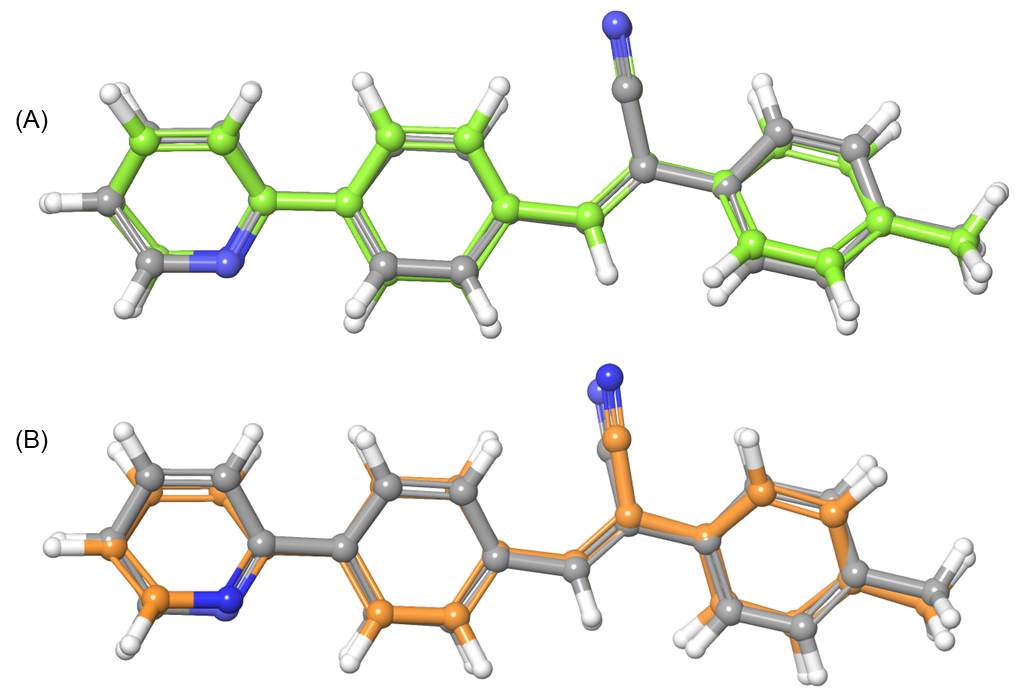
**

**Figure S10**. Structural overlay between X-ray and optimized structures of **2j** (A) molecule A and (B) molecule B. colour codes: grey (X-ray), green (optimized molecule A) and orange (optimized molecule B.

**Table S7**. Topological parameters for intramolecular interactions in **1** and **2j** at their (3, −1) BCPs. Definitions: *R*_ij_, bond path (Å); *ρ*(r), electron density (e Å^−3^); ∇^2^*ρ*(r), Laplacian of electron density (e Å^−5^); *V*(r), potential electron density (a.u.); *G*(r), kinetic electron density (a.u.); *H*(r), total electronic energy density (a.u.); *D*_e_, dissociation energy (kcal mol^−1^).

| Interaction | *R*_ij_ | *ρ*(r) | ∇^2^*ρ*(r) | *V*(r) | *G*(r) | *H*(r) | \|$\frac{-V(r)}{G(r)}$\| | *D*_e_ |
| --- | --- | --- | --- | --- | --- | --- | --- | --- |
| **Compound 1 (Mol A): X-ray** | | | | | | | | |
| H14A⋅⋅⋅C15A | 2.583 | 0.080 | 0.982 | -0.00724 | 0.008709 | 0.00147 | 0.83 | 2.3 |
| H8A⋅⋅⋅H2A | 2.146 | 0.096 | 1.268 | -0.00909 | 0.011123 | 0.00203 | 0.82 | 2.9 |
| H13A⋅⋅⋅H21A | 2.131 | 0.094 | 1.240 | -0.00878 | 0.010821 | 0.00204 | 0.81 | 2.8 |
| **Compound 1 (Mol A): optimized** | | | | | | | | |
| H14A⋅⋅C15A | 2.556 | 0.083 | 1.097 | -0.00822 | 0.0098 | 0.00159 | 0.84 | 2.6 |
| **Compound 1 (Mol B): X-ray** | | | | | | | | |
| H14B⋅⋅⋅C15B | 2.627 | 0.084 | 1.034 | -0.00755 | 0.00914 | 0.00159 | 0.83 | 2.4 |
| H13B⋅⋅⋅H21B | 2.136 | 0.094 | 1.236 | -0.00876 | 0.010793 | 0.00203 | 0.81 | 2.7 |
| H8B⋅⋅⋅H2B | 2.029 | 0.105 | 1.373 | -0.00996 | 0.012101 | 0.00214 | 0.82 | 3.1 |
| **Compound 1 (Mol B): optimized** | | | | | | | | |
| H14B⋅⋅⋅C15B | 2.557 | 0.095 | 1.160 | -0.00873 | 0.010384 | 0.00165 | 0.84 | 2.7 |
| **Compound 2j (Mol A): X-ray** | | | | | | | | |
| H14A⋅⋅⋅C15A | 2.616 | 0.080 | 0.978 | -0.0072 | 0.008669 | 0.00147 | 0.83 | 2.3 |
| H13A⋅⋅⋅H21A | 2.145 | 0.093 | 1.232 | -0.00874 | 0.010761 | 0.00202 | 0.81 | 2.7 |
| **Compound 2j (Mol A): optimized** | | | | | | | | |
| H14A⋅⋅⋅C15A | 2.540 | 0.092 | 1.125 | -0.00849 | 0.010081 | 0.0016 | 0.84 | 2.7 |
| **Compound 2j (Mol B): X-ray** | | | | | | | | |
| H14B⋅⋅⋅C15B | 2.579 | 0.083 | 1.024 | -0.00762 | 0.009118 | 0.0015 | 0.84 | 2.4 |
| H13B⋅⋅⋅H21B | 2.102 | 0.098 | 1.295 | -0.00926 | 0.01135 | 0.00209 | 0.82 | 2.9 |
| **Compound 2j (Mol B): optimized** | | | | | | | | |
| H14B⋅⋅⋅C15B | 2.560 | 0.093 | 1.141 | -0.00855 | 0.010195 | 0.00164 | 0.84 | 2.7 |


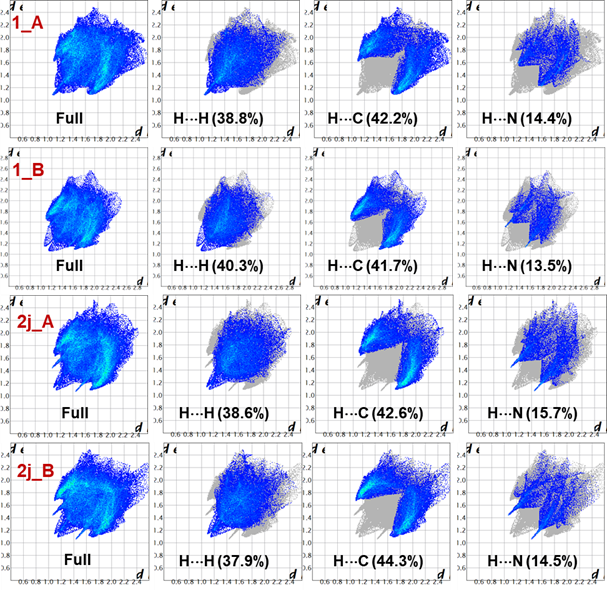


**Figure S11.** Full and decomposed fingerprint plots showing the relative contributions of important inter-contacts to the crystal packing of **1** and **2j**.


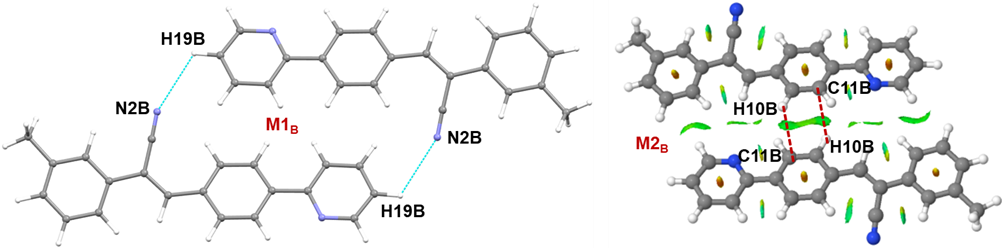


**Figure S12**. Molecular dimers (M1_B_ and M2_B_) formed between molecule B and its symmetry equivalent partners in **1**.

**Table S8**. Lattice energies (in kcal mol^-1^) for **1** and **2j**.

| Code | *E*_coul_ | *E*_pol_ | *E*_disp_ | *E*_rep_ | *E*_tot_ |
| --- | --- | --- | --- | --- | --- |
| **1** | -12.9 | -5.7 | -48.0 | -36.6 | -66.6 |
| **2j** | -13.3 | -5.5 | -47.9 | -36.3 | -66.7 |

**
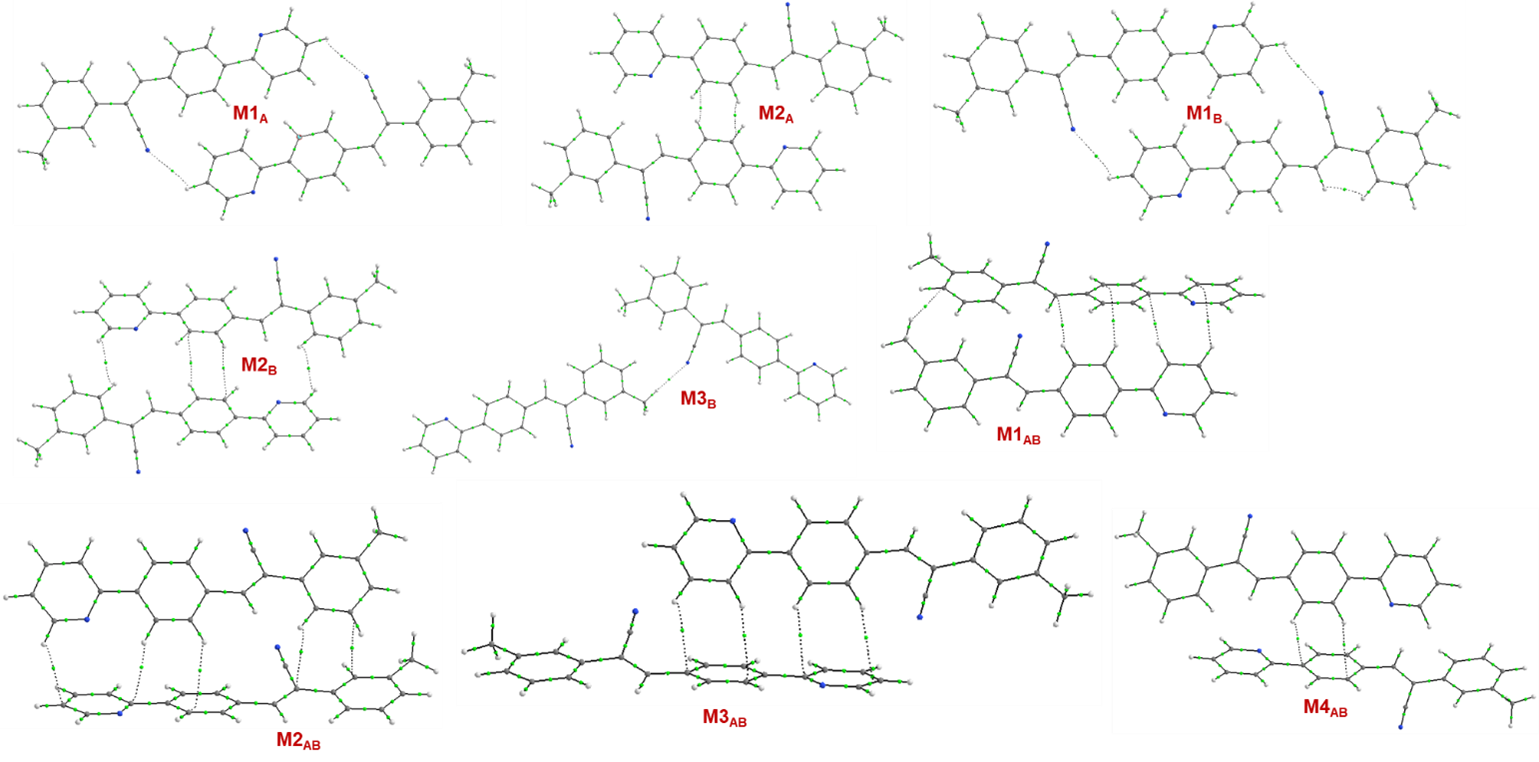
**

**Figure S13**. Molecular graphs of molecular dimers observed in **1** showing the bond critical points for intermolecular interactions.

**
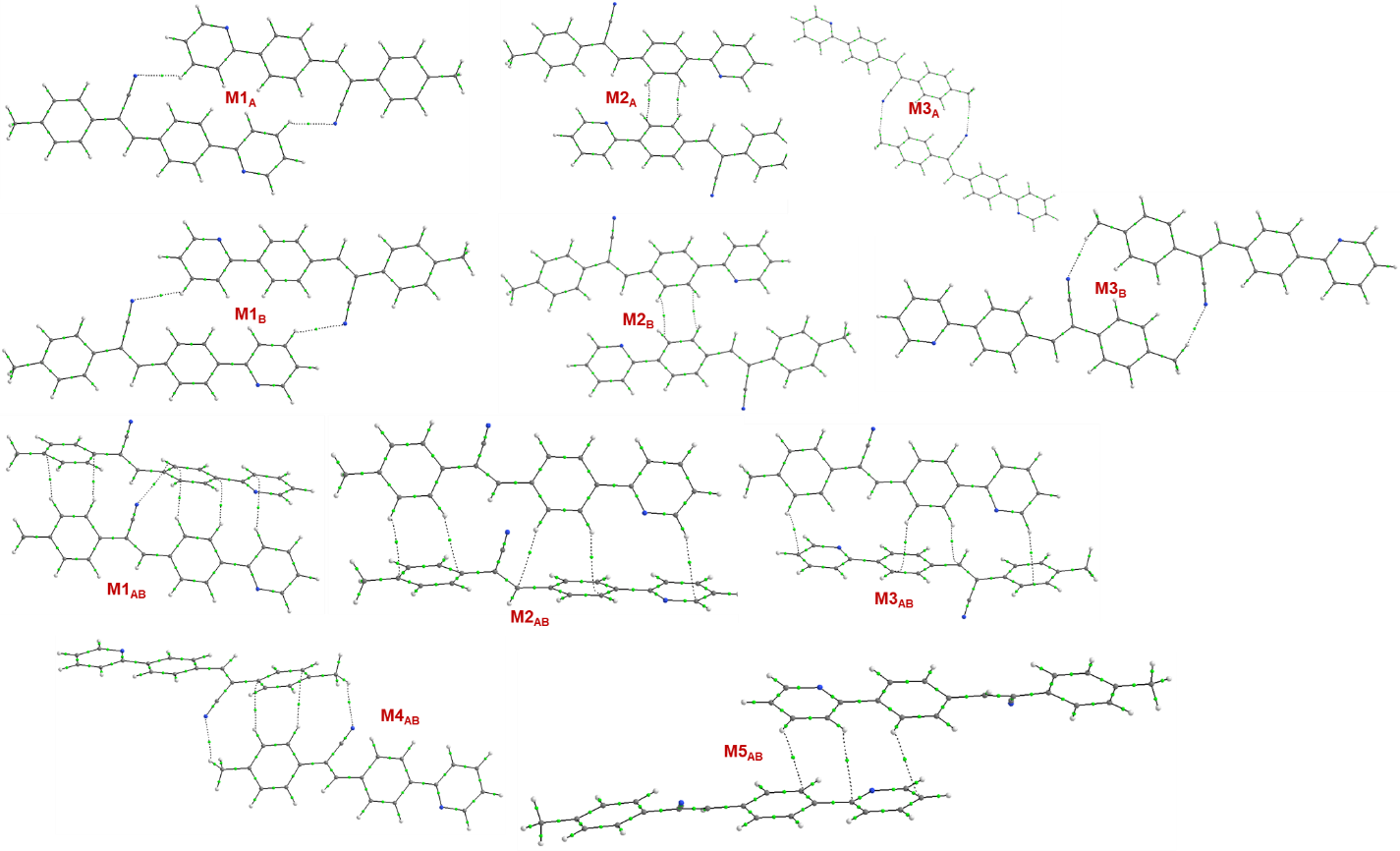
**

**Figure S14**. Molecular graphs of molecular dimers observed in **2j** showing the bond critical points for intermolecular interactions.

**Table S9**. Topological parameters for intermolecular interactions in dimers of **1** and **2j** at their (3, −1) BCPs. Definitions: *R*_ij_, bond path (Å); *ρ*(r), electron density (e Å^−3^); ∇^2^*ρ*(r), Laplacian of electron density (e Å^−5^); *V*(r), potential electron density (a.u.); *G*(r), kinetic electron density (a.u.); *H*(r), total electronic energy density (a.u.); *D*_e_, dissociation energy (kcal mol^−1^).

| Dimers | **Compound 1** | | | | | | | | |
| --- | --- | --- | --- | --- | --- | --- | --- | --- | --- |
|  | **Atoms** | *R*_ij_ | *ρ*(r) | ∇^2^*ρ*(r) | *V*(r) | *G*(r) | *H*(r) | De | \|$\frac{-V(r)}{G(r)}$\| |
| **M1_A_** | N2A···H19A | 2.858 | 0.038 | 0.495 | -0.00280 | 0.00397 | 0.00117 | 0.88 | 0.71 |
| **M2_A_** | C11A···H10A | 3.239 | 0.039 | 0.469 | -0.00273 | 0.00380 | 0.00107 | 0.86 | 0.72 |
| **M1_B_** | N2B···H19B | 2.941 | 0.033 | 0.414 | -0.00233 | 0.00331 | 0.00099 | 0.73 | 0.70 |
| **M2_B_** | H11B···C10B | 3.020 | 0.043 | 0.523 | -0.00313 | 0.00428 | 0.00115 | 0.98 | 0.73 |
| **M3_B_** | N2B···H22B | 2.667 | 0.044 | 0.560 | -0.00319 | 0.00450 | 0.00131 | 1.00 | 0.71 |
| **M1_AB_** | H21A···C12B | 2.751 | 0.049 | 0.566 | -0.00365 | 0.00476 | 0.00111 | 1.15 | 0.77 |
|  | H13A···C14B | 3.014 | 0.041 | 0.465 | -0.00285 | 0.00384 | 0.00099 | 0.89 | 0.74 |
|  | H14A···C8B | 2.905 | 0.038 | 0.442 | -0.00266 | 0.00362 | 0.00097 | 0.83 | 0.73 |
|  | H20A···C21B | 3.073 | 0.036 | 0.419 | -0.00250 | 0.00343 | 0.00092 | 0.79 | 0.73 |
|  | H222···C4B | 2.903 | 0.037 | 0.412 | -0.00249 | 0.00338 | 0.00089 | 0.78 | 0.74 |
| **M2_AB_** | H11A···C16B | 2.814 | 0.043 | 0.515 | -0.00320 | 0.00427 | 0.00107 | 1.00 | 0.75 |
|  | H10A···C11B | 2.985 | 0.040 | 0.467 | -0.00283 | 0.00384 | 0.00101 | 0.89 | 0.74 |
|  | H18A···C19B | 2.953 | 0.037 | 0.420 | -0.00254 | 0.00345 | 0.00091 | 0.80 | 0.74 |
|  | H2A···C8B | 3.122 | 0.033 | 0.396 | -0.00226 | 0.00318 | 0.00092 | 0.71 | 0.71 |
| **M3_AB_** | H21B···C12A | 2.880 | 0.046 | 0.519 | -0.00326 | 0.00432 | 0.00106 | 1.02 | 0.75 |
|  | H13B···C16A | 2.848 | 0.042 | 0.499 | -0.00317 | 0.00417 | 0.00101 | 0.99 | 0.76 |
|  | H20B···C9A | 2.920 | 0.041 | 0.476 | -0.00291 | 0.00392 | 0.00102 | 0.91 | 0.74 |
|  | H14B···C4A | 3.268 | 0.036 | 0.408 | -0.00241 | 0.00332 | 0.00091 | 0.76 | 0.73 |
| **M4_AB_** | C12A···H10B | 2.809 | 0.050 | 0.606 | -0.00384 | 0.00507 | 0.00122 | 1.21 | 0.76 |
|  | C14A···H11B | 2.808 | 0.046 | 0.529 | -0.00328 | 0.00439 | 0.00110 | 1.03 | 0.75 |
| **Compound 2j** | | | | | | | | | |
| **M1_A_** | N3A···H20A | 2.798 | 0.040 | 0.509 | -0.00296 | 0.00412 | -0.00116 | 0.93 | 0.72 |
| **M2_A_** | H10A···C11A | 3.447 | 0.046 | 0.568 | -0.00346 | 0.00468 | -0.00122 | 1.09 | 0.74 |
| **M3_A_** | H22B···N3A | 2.537 | 0.058 | 0.753 | -0.00446 | 0.00614 | -0.00168 | 1.40 | 0.73 |
| **M1_B_** | N3B···H20B | 2.842 | 0.038 | 0.477 | -0.00277 | 0.00386 | -0.00109 | 0.87 | 0.72 |
| **M2_B_** | H10B···C11 | 3.546 | 0.041 | 0.504 | -0.00295 | 0.00409 | -0.00114 | 0.92 | 0.72 |
| **M3_B_** | H22E···N3B | 2.523 | 0.060 | 0.783 | -0.00467 | 0.00640 | -0.00173 | 1.47 | 0.73 |
| **M1_AB_** | H14B···C14A | 2.983 | 0.043 | 0.490 | -0.00296 | 0.00402 | -0.00106 | 0.93 | 0.74 |
|  | H6B···C1A | 3.078 | 0.038 | 0.455 | -0.00266 | 0.00369 | -0.00103 | 0.84 | 0.72 |
|  | H13B···C12A | 2.959 | 0.037 | 0.431 | -0.00259 | 0.00353 | -0.00094 | 0.81 | 0.73 |
|  | H5B···C4A | 3.096 | 0.033 | 0.369 | -0.00218 | 0.00300 | -0.00083 | 0.68 | 0.73 |
| **M2_AB_** | C11A···H11B | 2.916 | 0.045 | 0.522 | -0.00321 | 0.00431 | -0.00111 | 1.01 | 0.74 |
|  | C8A···H10B | 2.893 | 0.042 | 0.495 | -0.00301 | 0.00407 | -0.00106 | 0.94 | 0.74 |
|  | C18A···H18B | 2.888 | 0.037 | 0.443 | -0.00276 | 0.00368 | -0.00092 | 0.87 | 0.75 |
|  | C4A···H3B | 2.985 | 0.036 | 0.403 | -0.00245 | 0.00332 | -0.00086 | 0.77 | 0.74 |
| **M3_AB_** | H10A···C13B | 3.106 | 0.047 | 0.537 | -0.00330 | 0.00443 | -0.00114 | 1.03 | 0.74 |
|  | H11A···C8B | 2.886 | 0.043 | 0.524 | -0.00317 | 0.00430 | -0.00113 | 0.99 | 0.74 |
|  | H3A···C19B | 2.995 | 0.039 | 0.439 | -0.00263 | 0.00359 | -0.00097 | 0.82 | 0.73 |
| **M4_AB_** | H22C···N3B | 2.692 | 0.044 | 0.545 | -0.00317 | 0.00441 | -0.00124 | 0.99 | 0.72 |
|  | N3A···H22E | 2.736 | 0.041 | 0.504 | -0.00295 | 0.00409 | -0.00114 | 0.92 | 0.72 |
|  | H5A···C1B | 3.011 | 0.038 | 0.453 | -0.00267 | 0.00369 | -0.00102 | 0.84 | 0.72 |
|  | H6A···C3B | 3.034 | 0.031 | 0.362 | -0.00211 | 0.00294 | -0.00083 | 0.66 | 0.72 |
| **M5_AB_** | H21A···C16B | 2.945 | 0.034 | 0.382 | -0.00236 | 0.00316 | -0.00081 | 0.74 | 0.75 |


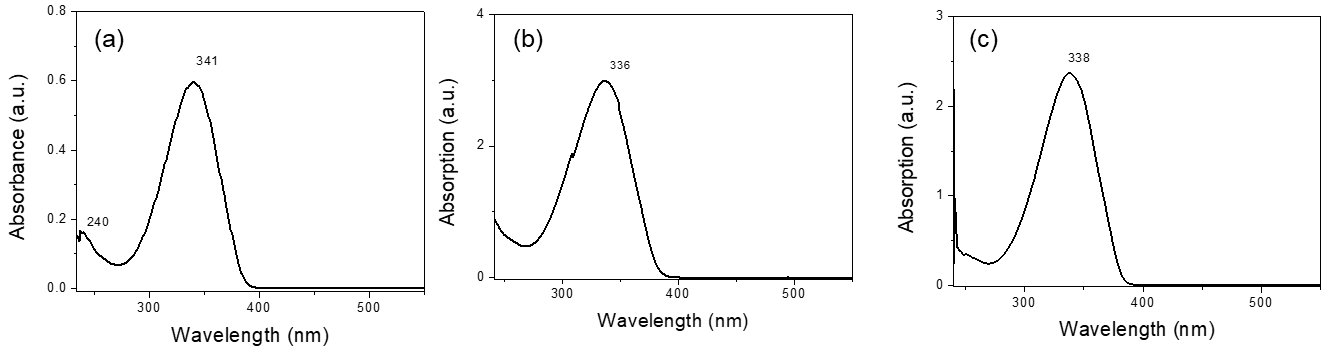


**Figure S15**. UV-vis absorption spectra for **1** in three different solvents **(A)** chloroform, **(B)** ethanol and **(C)** ethyl acetate


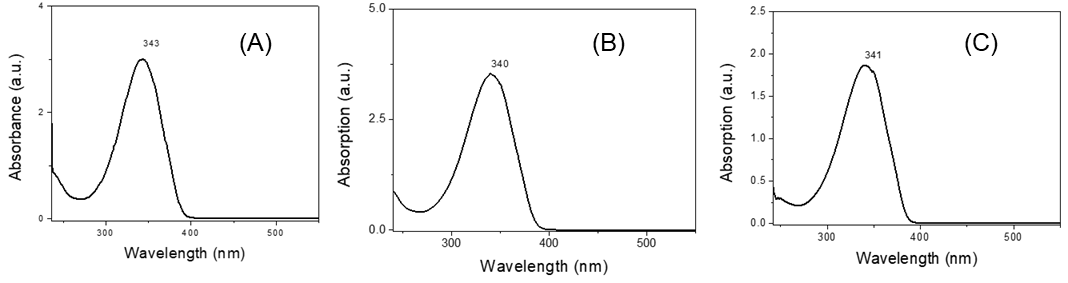


**Figure S16**. UV-vis absorption spectra for **2** in three different solvents **(A)** chloroform, **(B)** ethanol and **(C)** ethyl acetate

**Figure S17**. UV-vis absorption spectrum for **1** in the solid state.

**Figure S18**. UV-vis absorption spectrum for **2** in the solid state.

**Figure S19**. Tauc’s plot for **(A)** **1** and **(B)** **2** was obtained from absorption in chloroform.
